# Annexin A7 mediates lysosome repair independently of ESCRT-III

**DOI:** 10.3389/fcell.2023.1211498

**Published:** 2024-01-23

**Authors:** Malene Laage Ebstrup, Stine Lauritzen Sønder, Ditte Louise Fogde, Anne Sofie Busk Heitmann, Tiina Naumanen Dietrich, Catarina Dias, Marja Jäättelä, Kenji Maeda, Jesper Nylandsted

**Affiliations:** ^1^ Membrane Integrity, Danish Cancer Institute, Copenhagen, Denmark; ^2^ Cell Death and Metabolism, Danish Cancer Institute, Copenhagen, Denmark; ^3^ Bioimaging Core Facility, Danish Cancer Institute, Copenhagen, Denmark; ^4^ Department of Cellular and Molecular Medicine, Faculty of Health Sciences, University of Copenhagen, Copenhagen, Denmark; ^5^ Department of Molecular Medicine, University of Southern Denmark, Odense, Denmark

**Keywords:** lysosome membrane repair, lysosomal integrity, lysosomal membrane permeabilization, Annexin A7, endosomal sorting complexes required for transport III (ESCRT-III), L-Leucyl-L-Leucine O-methyl ester (LLOMe), organelle repair, ER-lysosome membrane contact sites (MCSs)

## Abstract

Lysosomes are crucial organelles essential for various cellular processes, and any damage to them can severely compromise cell viability. This study uncovers a previously unrecognized function of the calcium- and phospholipid-binding protein Annexin A7 in lysosome repair, which operates independently of the Endosomal Sorting Complex Required for Transport (ESCRT) machinery. Our research reveals that Annexin A7 plays a role in repairing damaged lysosomes, different from its role in repairing the plasma membrane, where it facilitates repair through the recruitment of ESCRT-III components. Notably, our findings strongly suggest that Annexin A7, like the ESCRT machinery, is dispensable for membrane contact site formation within the newly discovered phosphoinositide-initiated membrane tethering and lipid transport (PITT) pathway. Instead, we speculate that Annexin A7 is recruited to damaged lysosomes and promotes repair through its membrane curvature and cross-linking capabilities. Our findings provide new insights into the diverse mechanisms underlying lysosomal membrane repair and highlight the multifunctional role of Annexin A7 in membrane repair.

## Introduction

Lysosomes are the major degradative compartments of eukaryotic cells and participate in the degradation and recycling of intracellular and extracellular material (Saftig and Klumperman, 2009; Hesketh et al., 2018; Ballabio and Bonifacino, 2020). The degradative activity of lysosomes originates from their low intralumenal pH and high content of hydrolases. Consequently, physiological, and pathological inducers of lysosomal membrane permeabilization (LMP) lead to the release of hydrolases from the lysosomal lumen into the cytosol, severely threatening cell viability (Kroemer and Jäättelä, 2005; Boya and Kroemer, 2008; Aits and Jäättelä, 2013; Papadopoulos and Meyer, 2017; Wang et al., 2018). Thus, cells must cope with lysosome damage by repairing or disposing of damaged lysosomes to avert LMP-induced cell death.

The cell can dispose of damaged lysosomes through a selective type of autophagy termed lysophagy (Hung et al., 2013; Maejima et al., 2013; Papadopoulos and Meyer, 2017; Papadopoulos et al., 2020). In this pathway, intralumenal carbohydrate chains of glycoproteins of autophagy-targeted lysosomes are bound and ubiquitinated by cytosolic lectins and ubiquitin ligases, such as galectin-3 (GAL3) and tripartite motif-containing protein 16 (TRIM16) (Paz et al., 2010; Thurston et al., 2012; Hung et al., 2013; Maejima et al., 2013; Chauhan et al., 2016; Papadopoulos and Meyer, 2017; Yoshida et al., 2017; Koerver et al., 2019; Jia et al., 2020; Papadopoulos et al., 2020). Subsequent processing of ubiquitin chains by ATPase 97 enables microtubule-associated protein 1A/1B-light chain 3 (LC3)-containing autophagic membranes to associate with damaged lysosomes promoting their degradation (Fujita et al., 2013; Hung et al., 2013; Papadopoulos et al., 2017; Papadopoulos and Meyer, 2017; Koerver et al., 2019; Papadopoulos et al., 2020). However, recent studies have shown that damaged lysosomes can be salvaged from lysophagy by activating endogenous repair mechanisms (Scharf et al., 2012; Radulovic et al., 2018; Skowyra et al., 2018; Niekamp et al., 2022; Radulovic et al., 2022; Tan and Finkel, 2022; Yim et al., 2022). Recruitment of the Endosomal Sorting Complex Required for Transport (ESCRT) machinery to damaged endolysosomal compartments promotes repair and cell survival (Radulovic et al., 2018; Skowyra et al., 2018). ESCRT proteins are categorized into four subcomplexes designated ESCRT-0, -I, -II and–III, of which the assembly of ESCRT-III into a spiral-shaped oligomeric structure mediates membrane sealing/scission in a wide variety of cellular processes such as cytokinetic abscission, intralumenal vesicle (ILV) biogenesis and repair (Vietri et al., 2020).

In lysosome repair, the calcium sensor and ESCRT-III binding protein, apoptosis-linked gene-2 (ALG-2) interacting protein X (ALIX), and the ESCRT-I component, tumor susceptibility gene 101 (TSG101), cooperate to recruit ESCRT-III to the damaged compartments, where it facilitates repair through a currently unknown mechanism. Importantly, ESCRT-mediated lysosome repair occurs before and independent of the lysophagic pathway, as the ESCRT-III component, charged multivesicular body protein 4B (CHMP4B), is recruited to damaged compartments prior to prominent lysophagic markers (e.g., GAL3, ubiquitin and LC3) (Radulovic et al., 2018), and its recruitment is unaffected by inhibition of the lysophagic pathway (Radulovic et al., 2018; Skowyra et al., 2018).

In addition to the ESCRT machinery, alternative ESCRT-independent lysosome repair mechanisms have recently been identified. One of the mechanisms proposed for lysosome repair involves calcium-activated sphingomyelin scrambling and turnover, which leads to the formation of ceramide microdomains on the cytosolic surface of the damaged lysosomes, potentially facilitating ESCRT-independent repair through the induction of negative membrane curvature (Niekamp et al., 2022). Moreover, a phosphoinositide-initiated membrane tethering and lipid transport (PITT) pathway has been discovered, promoting lysosome repair via ER-mediated phosphatidylserine (PS) and cholesterol transfer, achieved through extensive ER-lysosomal membrane contact sites (MCSs) (Radulovic et al., 2022; Tan and Finkel, 2022). Finally, Annexins (ANXAs) have also been associated with lysosome repair (Scharf et al., 2012; Yim et al., 2022). Members of the ANXA protein family are calcium-sensitive and phospholipid-binding proteins that regulate various endomembrane processes, including membrane segregation, compartmentalization, and vesicle trafficking and fusion (Hayes et al., 2004; Draeger et al., 2011; Boye and Nylandsted, 2016). Furthermore, ANXAs are important components of the plasma membrane repair system (Draeger et al., 2011; Boye and Nylandsted, 2016; Boye et al., 2017; Sønder et al., 2019). All ANXAs contain a conserved C-terminal core domain, which contains the type II calcium-binding sites that enable ANXAs to bind anionic phospholipids in membranes in a reversible calcium-dependent manner (Blackwood and Ernst, 1990; Gerke and Moss, 2002). Two members of the ANXA family, ANXA1 and ANXA2, have been directly linked to lysosome repair (Scharf et al., 2012; Yim et al., 2022). ANXA2 and its binding partner S100A10 have been shown to be recruited to late endosomal and lysosomal membranes in dendritic cells during organelle destabilization following phagocytosis of polyethylene particles (Scharf et al., 2012). Here, ANXA2 is suggested to participate in stabilizing or resealing damaged membranes, thereby reducing the innate inflammatory response. Both ANXA1 and ANXA2 have been shown to facilitate lysosome repair in human osteosarcoma U2OS cells (Yim et al., 2022). These ANXAs predominantly translocate to and promote the repair of larger lysosomal lesions (>4.6 nm), likely complementing ESCRT-mediated repair, as lysosomes that were positive for ANXA1/2 were also recurrently positive for the ESCRT-III component CHMP4A (Yim et al., 2022). These recent discoveries highlight the importance of lysosome integrity in cellular health and suggest that lysosome repair, like plasma membrane repair, is a dynamic multi-layered process with different independent mechanisms complimenting each other.

We have previously demonstrated that ANXA7 is required for recruitment and ESCRT-III-mediated repair at the plasma membrane (Sønder et al., 2019). Following plasma membrane injury, calcium-mediated recruitment of ANXA7 enables complex formation with ALG-2 (or PDCD6), facilitating ALIX recruitment and binding. Together ALG-2 and ALIX mediate subsequent ESCRT-III assembly and shedding of the damaged membrane portion (Sønder et al., 2019). The mechanistic overlap between repair at the plasma membrane and endolysosomal compartments questions whether ANXA7, and possibly other ANXAs, may also be involved in lysosome repair and whether such a potential role depends on the ESCRT machinery.

Our findings provide compelling evidence that ANXA7 plays a crucial role in lysosome repair, and this role is mechanistically distinct from its established function in plasma membrane repair, where it operates through the recruitment of ESCRT-III components. This discovery unveils a novel and previously unrecognized function of ANXA7 in membrane repair. Furthermore, our data strongly indicate that ANXA7, similar to the ESCRT machinery, is dispensable for lipid transfer through the newly discovered PITT pathway.

## Results

### Endogenous Annexin A7 co-purifies with lysosomes following LLOMe treatment

**FIGURE 1:**
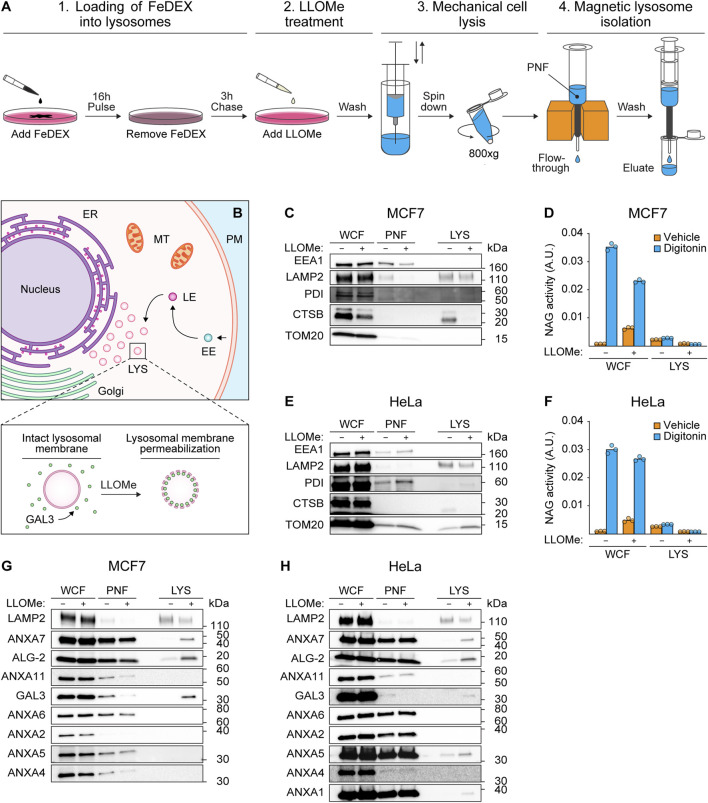
ANXA7 co-purifies with lysosomal markers following treatment with LLOMe. **(A)** Workflow of magnetic isolation of FeDEX-loaded lysosomes from MCF7 and HeLa cells using a Pulse-Chase approach. 1) Addition of FeDEX (40 kDa) to the cell culture medium for 16 h (Pulse) followed by 3 h in clean culture medium (Chase). 2) Cells were treated with vehicle (diH_2_O) or LLOMe (2 mM) during the last hour of the chase period before harvest, and 3) mechanical cell lysis. 4) Magnetic retention and subsequent elution of FeDEX-loaded lysosomes. The figure is adapted from Bilgin et al. (2017). **(B)** Lysosomes constitute one of many intracellular organelles. Treatment with LLOMe induces LMP within cells, permitting the binding of damage marker GAL3 to exposed intralumenal β-galactosides. **(C,E,G, and H)** Immunoblot of WCF, PNF and LYS fractions from cells treated with vehicle (diH_2_O) or LLOMe (2 mM) for 1 h showing bands detected with antibodies against the indicated cellular compartments. Original unedited blots are presented in Supplementary Figure S1. **(D,F)** NAG activity of WCF and LYS fractions. The NAG activity was used as an indirect measure of LLOMe-induced LMP using the membranolytic agent, digitonin, as a positive control. The presented data constitutes a single biological replicate from the cellular fractions blotted in **(C,E,G, and H)**. Data points reflect technical replicates. The remaining biological replicates are presented in Supplementary Figures S2A, B. FeDEX-based lysosomal isolation was performed once for HeLa and thrice for MCF7 cells. Abbreviations: (ALG-2) apoptosis-linked gene-2, (ANXA) annexin, (CTSB) cathepsin B, (EE) early endosome, (EEA1) early endosome antigen 1, (ER) endoplasmic reticulum, (FeDEX) superparamagnetic colloidal iron dextran, (GAL3) galectin-3, (LAMP2) lysosome-associated membrane glycoprotein 2, (LE) late endosome, (LMP) lysosomal membrane permeabilization, (LLOMe) L-Leucyl-L-Leucine *O*-methyl ester, (LYS) lysosome, (MT) mitochondria, (PM) plasma membrane, (PNF) post-nuclear fraction, (NAG) N-acetyl-β-D-glucosaminidase, (PDI) protein disulfide-isomerase, (TOM20) mitochondrial import receptor subunit TOM20, (WCF) whole-cell fraction.

To investigate whether ANXA7 participates in lysosome repair, we treated MCF7 breast carcinoma cells and HeLa cervix carcinoma cells with L-Leucyl-L-Leucine *O*-methyl ester (LLOMe), a lysosomotropic agent which enters cells via receptor-mediated endocytosis and is converted to a membranolytic compound specifically in the endolysosomal compartments (Thiele and Lipsky, 1990). Prior to LLOMe treatment (1 h), lysosomes of MCF7 or HeLa cells were loaded with superparamagnetic colloidal iron dextran (FeDEX) (16 h), enabling magnetic retention and subsequent elution and characterization of FeDEX-loaded lysosomes by immunoblotting (Figure 1A).

The FeDEX-based isolation procedure successfully enriched lysosomal markers, lysosome-associated membrane glycoprotein 2 (LAMP2) and cathepsin B (CTSB), whereas cellular markers for early endosomes (EEA1), the ER (PDI) and mitochondria (TOM20) were vastly depleted (Figures 1B, C, E; Supplementary Figure S1). Notably, LLOMe treatment depleted the lysosomal protease, CTSB, in the lysosomal fraction, suggestive of LLOMe-induced LMP and loss of lysosomal content. Accordingly, treatment with LLOMe reduced the extent to which the pore-forming detergent, digitonin, could release N-acetyl-β-D-glucosaminidase (NAG) from lysosomes in both whole-cell and lysosomal fractions observable as reduced NAG activity (Figures 1D, F). Collectively, the data demonstrate that the procedure successfully isolates lysosomes and treatment with LLOMe effectively induces LMP.

Prompted by our previous studies of ANXAs and their roles in plasma membrane repair (Lauritzen et al., 2015; Boye and Nylandsted, 2016; Boye et al., 2017; Sønder et al., 2019; Bendix et al., 2020; Simonsen et al., 2020; Moreno-Pescador et al., 2022), we wondered if similar repair mechanisms might occur on lysosomal membranes upon damage. To confirm lysosomal damage, we blotted against GAL3 as a sensitive marker of LMP due to its rapid translocation to leaky lysosomes in response to LLOMe treatment (Figure 1B) (Aits et al., 2015). Interestingly, while the lysosomal fraction from LLOMe-treated HeLa and MCF7 cells showed enriched GAL3 levels as expected, we also detected an enrichment of both ANXA7 and ALG-2 (Figures 1G, H; Supplementary Figure S1). Moreover, we detected an enrichment of ANXA1 and ANXA5 in the lysosomal fraction from LLOMe-treated HeLa cells, whereas ANXA2, ANXA4, and ANXA6 were absent in the lysosomal fractions from both cell lines (Figures 1G, H; Supplementary Figure S1). These findings indicate that, despite their conserved structure and functional similarities, ANXAs have distinct cellular functions that are cell type specific. Moreover, the data suggest that endogenous ANXA7 and ALG-2 are recruited to damaged lysosomes in both MCF7 and HeLa cells. Based on this and our prior demonstration of ANXA7’s necessity for ESCRT-III-mediated plasma membrane repair, we chose to focus on investigating the potential role of ANXA7 in lysosome damage and repair.

### ANXA7 translocates to GAL3-positive vesicles independently of ALG-2

**FIGURE 2:**
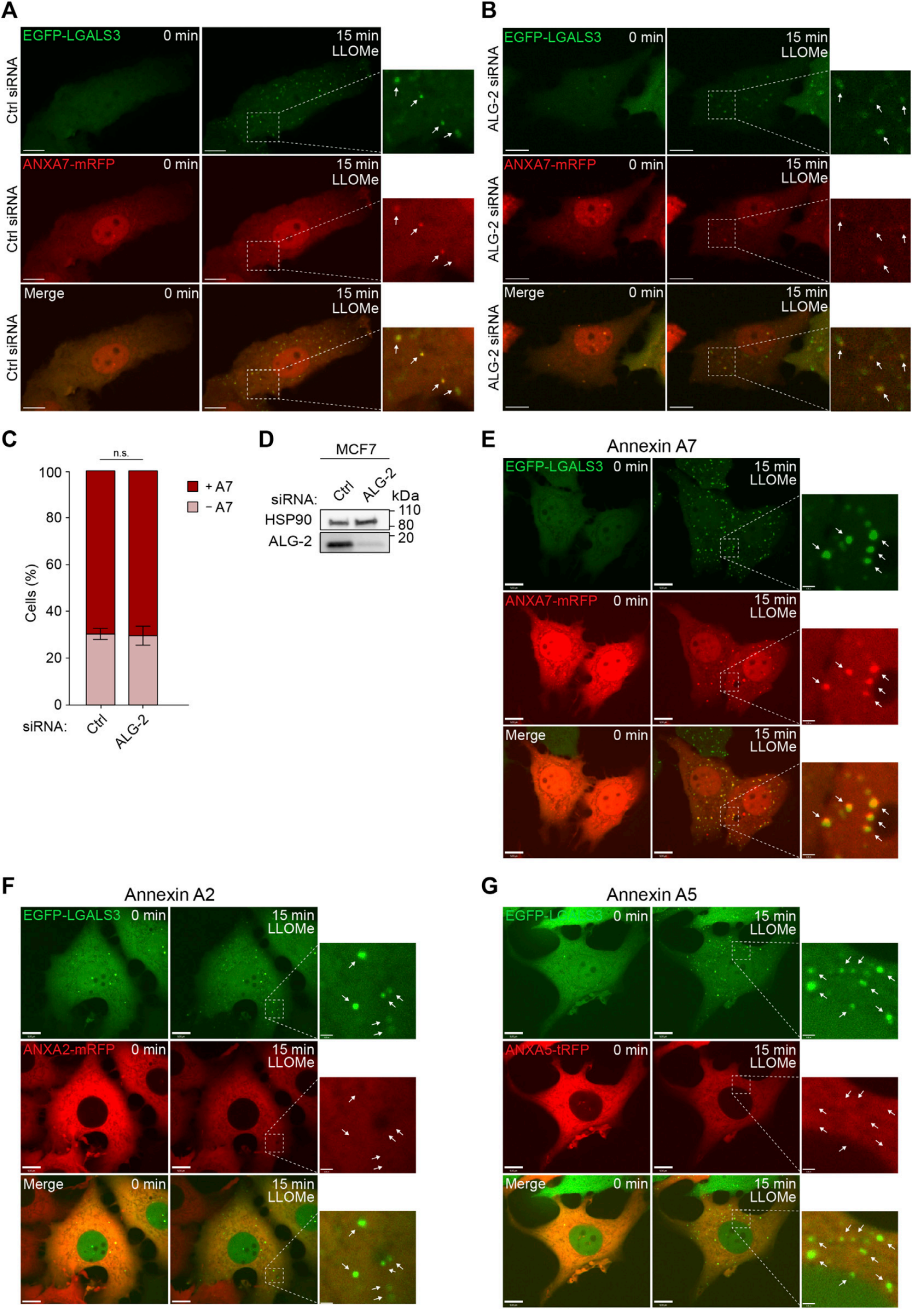
ANXA7 translocates to GAL3-positive vesicles following LLOMe-induced lysosomal membrane permeabilization independently of ALG-2. **(A,B)** Representative images of ANXA7 and GAL3 puncta formation upon exposure to LLOMe (5 mM) in MCF7 cells transiently co-expressing EGFP-LGALS3 and ANXA7-mRFP. Cells were transfected with Ctrl or ALG-2 siRNA 72 h prior to exposure to LLOMe. White arrows show GAL3 and ANXA7 puncta in zoomed-in images. Scale bar **(A,B)** denotes 10 µm. The image intensities have been adjusted for improved visual presentation. Supplementary Movies S1, S2 of the image sequences is presented in the Supplementary Material. **(C)** Percentage of MCF7 cells positive or negative for ANXA7 puncta formation in response to LLOMe (5 mM) and pretreatment with Ctrl or ALG-2 siRNA. Cells were quantified as positive if displaying 5 ≥ newly formed ANXA7 puncta within 15 min of LLOMe treatment. Data from four independent experiments are quantified from ≥13 cells per condition and depicted as mean ± SD. The *p*-value was defined by an unpaired two-tailed *t*-test with Welch’s correction. **(D)** Corresponding representative immunoblot of lysates after siRNA transfection (72 h) showing bands detected with antibodies against ALG-2 and HSP90 (loading control). Original unedited blots are presented in Supplementary Figure S3. **(E–G)** Representative images of GAL3 and ANXA7 puncta formation upon exposure to LLOMe (5 mM) in MCF7-EGFP-LGALS3 cells transiently co-expressing ANXA2-mRFP, ANXA5-tRFP or ANXA7-mRFP. White arrows show GAL3 and ANXA7 puncta in zoomed-in images. The image intensities have been adjusted for improved visual presentation. Scale bars **(E–G)** denote 1.10 and 9.00 µm, respectively. Supplementary Movies S3–S5 of the image sequences is presented in the Supplementary Material. Abbreviations: same abbreviations as in Figure 1.

To further validate our data, we used MCF7 cells stably expressing GAL3 fused to enhanced green fluorescent protein (MCF7 EGFP-LGALS3) as a well-established and sensitive marker to identify and study lysosome damage and repair in live cells (Aits et al., 2015). Cells co-expressing ANXA7 fused to monomeric red fluorescent protein (ANXA7-mRFP) were exposed to LLOMe and imaged for 15 min by time-lapse microscopy. The cells showed strong GAL3 puncta formation upon LLOMe treatment, as expected and previously demonstrated (Aits et al., 2015). In addition, LLOMe treatment triggered pronounced ANXA7 puncta formation at vesicles, becoming positive for GAL3 (Figure 2E; Supplementary Movie S3), in agreement with our lysosomal fractionation data (Figures 1G, H; Supplementary Figure S1). Cell death assays were performed to ascertain that LLOMe did not impact the short-term cell viability in the applied time frame and concentration range, which was not the case (Supplementary Figures S2C–G).

From the FeDEX-based isolation procedure of lysosomes, we detected an enrichment of ANXA7 and ALG-2 in both the lysosomal fractions from LLOMe-treated MCF7 and HeLa cells (Figures 1G, H; Supplementary Figure S1). We have previously shown that ANXA7 and ALG-2 form a complex upon repair at the plasma membrane and that ANXA7 binding to free membrane edges is independent of ALG-2, whereas ANXA7 is required for ALG-2 membrane binding and positioning at the site of repair (Sønder et al., 2019). To validate whether ANXA7 translocation to damaged lysosomes is independent of ALG-2, we investigated the translocation kinetics of ANXA7-mRFP following LLOMe treatment in control and ALG-2 siRNA depleted MCF7 cells. The cells were co-transfected with EGFP-GAL3 to verify lysosomal damage after LLOMe treatment. The siRNA transfected MCF7 cells co-expressing EGFP-GAL3 and ANXA7-mRFP were exposed to LLOMe and imaged for 15 min by time-lapse confocal microscopy. LLOMe treatment induced accumulation of both GAL3 and ANXA7 puncta (Figures 2A, B; Supplementary Movies S1, S2). However, siRNA depletion of ALG-2 (>90%) did not significantly decrease ANXA7 puncta after LLOMe treatment as compared to control siRNA transfected cells, indicating that ANXA7 translocates to damaged lysosomes independently of ALG-2 (Figures 2A–D; Supplementary Figure S3A; Supplementary Movies S1, S2).

To address whether the observed damage response is specific to ANXA7, we also investigated the translocation kinetics of other ANXAs in response to LLOMe treatment. We chose to include ANXA2 and ANXA5 as the most auspicious candidates, as ANXA2 has previously been described to bind damaged endosomes and to facilitate lysosome repair (Scharf et al., 2012; Yim et al., 2022), while we detected an enrichment of ANXA5 in the lysosomal fraction from LLOMe-treated HeLa cells (Figure 1H). As before, LLOMe treatment induced accumulation of GAL3 puncta. However, neither ANXA2-mRFP nor ANXA5 fused to turbo-RFP (ANXA5-tRFP) formed any puncta in response LLOMe treatment (Figures 2F, G; Supplementary Movies S4, S5). Thus, there seems to be a specificity of ANXA7 towards damaged lysosomes in MCF7 cells compared to ANXA2 and ANXA5.

### ANXA7 knockout sensitizes cells to LLOMe-induced lysosomal membrane permeabilization independently of ESCRT-III recruitment

**FIGURE 3:**
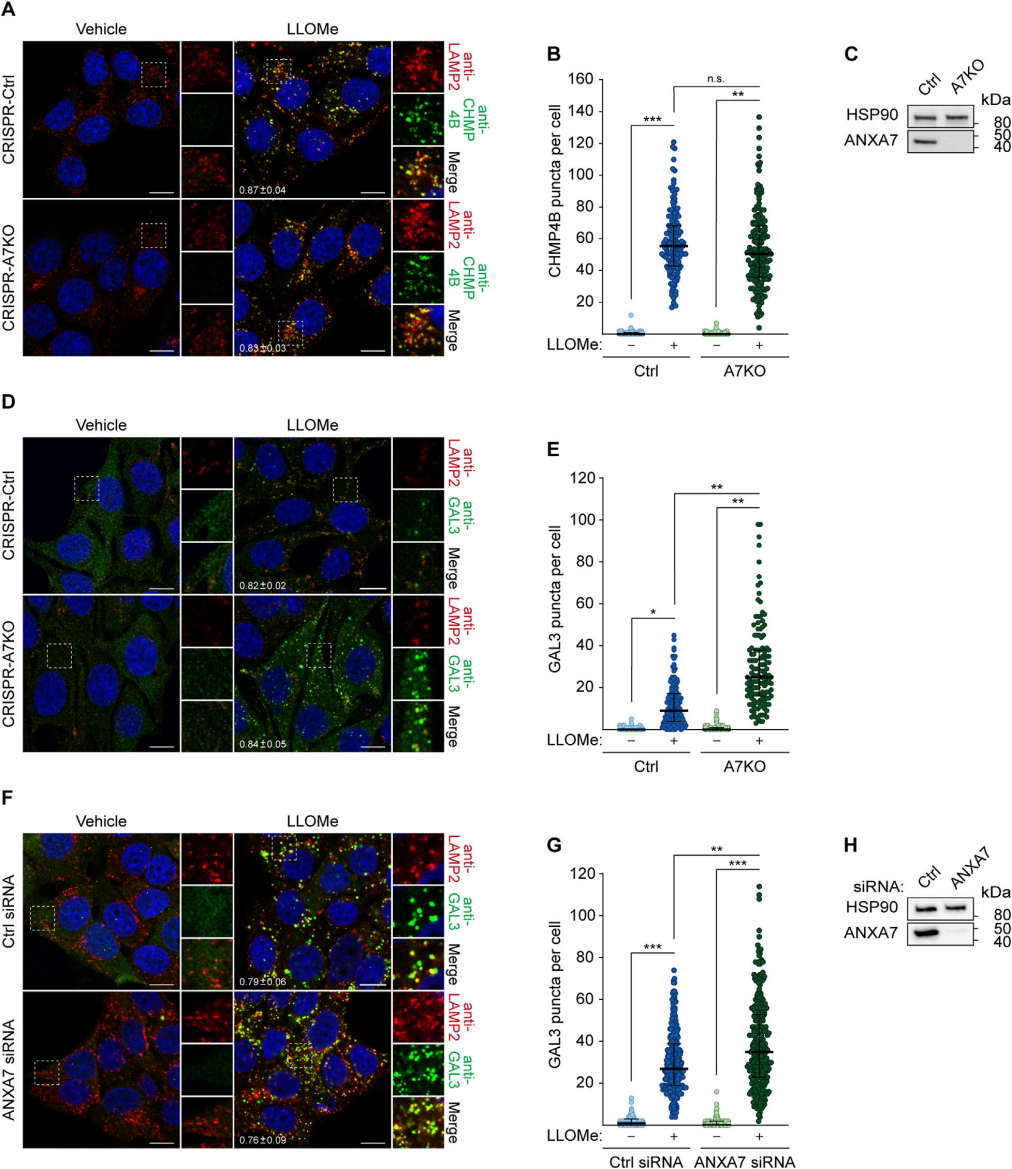
ANXA7 knockout sensitizes MCF7-p95ErbB2 cells to LLOMe-induced lysosomal membrane permeabilization independently of ESCRT-III recruitment. **(A)** Representative images of MCF7-p95ErbB2 CRISPR-A7KO and CRISPR-Ctrl cells treated with vehicle (diH_2_O) or LLOMe (2 mM, 30 min) prior to fixation and immunostaining with Hoechst 33342 (blue), anti-CHMP4B (green) and anti-LAMP2 (red). **(B)** CHMP4B puncta per cell quantified based on the data described in **(A)**. Data from three independent experiments are quantified from ≥42 cells per condition and depicted as single observations, median and interquartile range. **(C)** Immunoblot of MCF7-p95ErbB2 CRISPR-A7KO and CRISPR-Ctrl lysates showing bands detected with antibodies against ANXA7 and HSP90 (loading control). **(D)** Representative images of MCF7-p95ErbB2 CRISPR-A7KO and -Ctrl cells treated with vehicle (diH_2_O) or LLOMe (2 mM, 30 min) prior to fixation and immunostaining with Hoechst (blue), anti-GAL3 (green) and anti-LAMP2 (red). **(E)** GAL3 puncta per cell quantified based on the data described in **(D)**. Data from three independent experiments are quantified from ≥26 cells per condition and depicted as single observations, median and interquartile range. **(F)** Representative images of MCF7 cells transfected with ANXA7 or Ctrl siRNA (72 h) and treated with vehicle (diH_2_O) or LLOMe (2 mM, 30 min) prior to fixation and immunostaining with Hoechst 33342 (blue), anti-GAL3 (green) and anti-LAMP2 (red). **(G)** GAL3 puncta per cell quantified based on the data described in **(F)**. Data from three independent experiments are quantified from ≥64 cells per condition and depicted as single observations, median and interquartile range. **(H)** Immunoblot of MCF7 showing bands detected with antibodies against ANXA7 and HSP90 (loading control). The mean Manders’ overlap coefficient ± SD for LLOMe treated conditions are presented in the bottom left corner of each image **(A,D, and F)**. Scale bar denotes 10 µm. The *p*-values were defined by an unpaired two-tailed *t*-test with Welch’s correction comparing the mean values between the indicated conditions. *p ≤ 0.05; **p ≤ 0.01; ***p ≤ 0.001. Original unedited blots are presented in Supplementary Figures S5A, B. Abbreviations: (CHMP4B) charged multivesicular body protein 4B, (HSP90) heat shock protein 90. Remaining abbreviations as in Figure 1.

In light of our earlier findings demonstrating the requirement of ANXA7 for ESCRT-III-mediated plasma membrane repair (Sønder et al., 2019), we wondered if ANXA7 likewise promotes ESCRT-III-mediated lysosome repair. We approached this question using our previously generated MCF7-p95ErbB2 cells with CRISPR/Cas9 disrupted ANXA7 gene expression (CRISPR-A7KO) (Sønder et al., 2019), which was used to investigate the role of ANXA7 in plasma membrane repair. First, we treated MCF7-p95ErbB2 CRISPR-A7KO and control (CRISPR-Ctrl) cells with LLOMe or vehicle (diH_2_O) (30 min) before fixation and immunostaining with anti-CHMP4B as a marker for the ESCRT-III complex. LLOMe treatment induced massive CHMP4B puncta formation co-occurring with LAMP2 puncta, strongly indicating lysosomal recruitment of the ESCRT-III complex as expected and previously reported (Radulovic et al., 2018; Skowyra et al., 2018; Niekamp et al., 2022; Yim et al., 2022). Surprisingly, we detected no significant difference in the number of CHMP4B puncta between CRISPR-A7KO and CRISPR-Ctrl cells (Figures 3A–C; Supplementary Figure S5A). These data show ANXA7 to be dispensable for CHMP4B buildup and ESCRT-III assembly at the lysosomal membrane in MCF7-p95ErbB2 cells, demonstrating that the damage response of ANXA7 at the plasma membrane and endolysosomal compartments differs mechanistically.

Next, we opted to investigate whether ANXA7 knockout impacted lysosome damage by treating MCF7-p95ErbB2 CRISPR-A7KO and CRISPR-Ctrl cells with LLOMe or vehicle (30 min) prior to fixation and immunostaining with anti-GAL3. LLOMe treatment triggered massive GAL3 puncta formation co-occurring with LAMP2 puncta (Figures 3D, E), highly indicative of lysosomal recruitment of GAL3 as expected and previously reported (Aits et al., 2015). Interestingly, CRISPR-A7KO cells displayed significantly more GAL3 puncta per cell than CRISPR-Ctrl cells after LLOMe treatment (Figures 3D, E), suggesting that ANXA7 knockout sensitizes MCF7-p95ErbB2 cells to LLOMe-induced LMP independently of ESCRT-III recruitment. Importantly, during steady-state conditions (vehicle treatment), no difference in GAL3 accumulation was detected between the cell lines (Figures 3D, E), indicating that ANXA7 specifically functions in response to provoked and acute lysosome damage. Following this discovery, we repeated the setup and included a siRNA targeting CHMP4B to evaluate the impact of disrupted ESCRT-III-mediated lysosome repair on GAL3 puncta formation (Supplementary Figures S4A–C, S5C, D). SiRNA depletion of CHMP4B (>90%) did not significantly alter GAL3 accumulation in the CRISPR-Ctrl cells as compared to control siRNA transfected cells (Supplementary Figures S4A–C, S5C, D), consistent with previous demonstrations by Skowyra et al. (2018). However, we were able to reproduce the increase in GAL3 accumulation upon ANXA7 knockout for both siRNA conditions (Ctrl and CHMP4B#2 siRNA) (Supplementary Figures S4A, B), reaffirming the detrimental impact that ANXA7 knockout has on the lysosomal damage response.

SiRNA depletion of ANXA7 (>90%) significantly enhanced GAL3 accumulation upon LLOMe treatment in MCF7 cells as compared to control siRNA transfected cells (Figures 3F–H; Supplementary Figure S5B), showing that ANXA7 depletion robustly sensitizes MCF7 cells and their derived cell lines to LLOMe-induced LMP. Furthermore, this demonstrates that the observed increase in GAL3 accumulation is not an outcome of single cell-derived knockout clones or heightened membrane dynamics and invasiveness of the MCF7-p95ErbB2 cell model (Martin et al., 2012; Rafn et al., 2012). Collectively, these data demonstrate that lack of ANXA7 negatively impacts lysosome repair in MCF7 cells in a manner mechanistically distinct from the ESCRT machinery.

### ANXA7 knockout impairs lysosome repair

**FIGURE 4:**
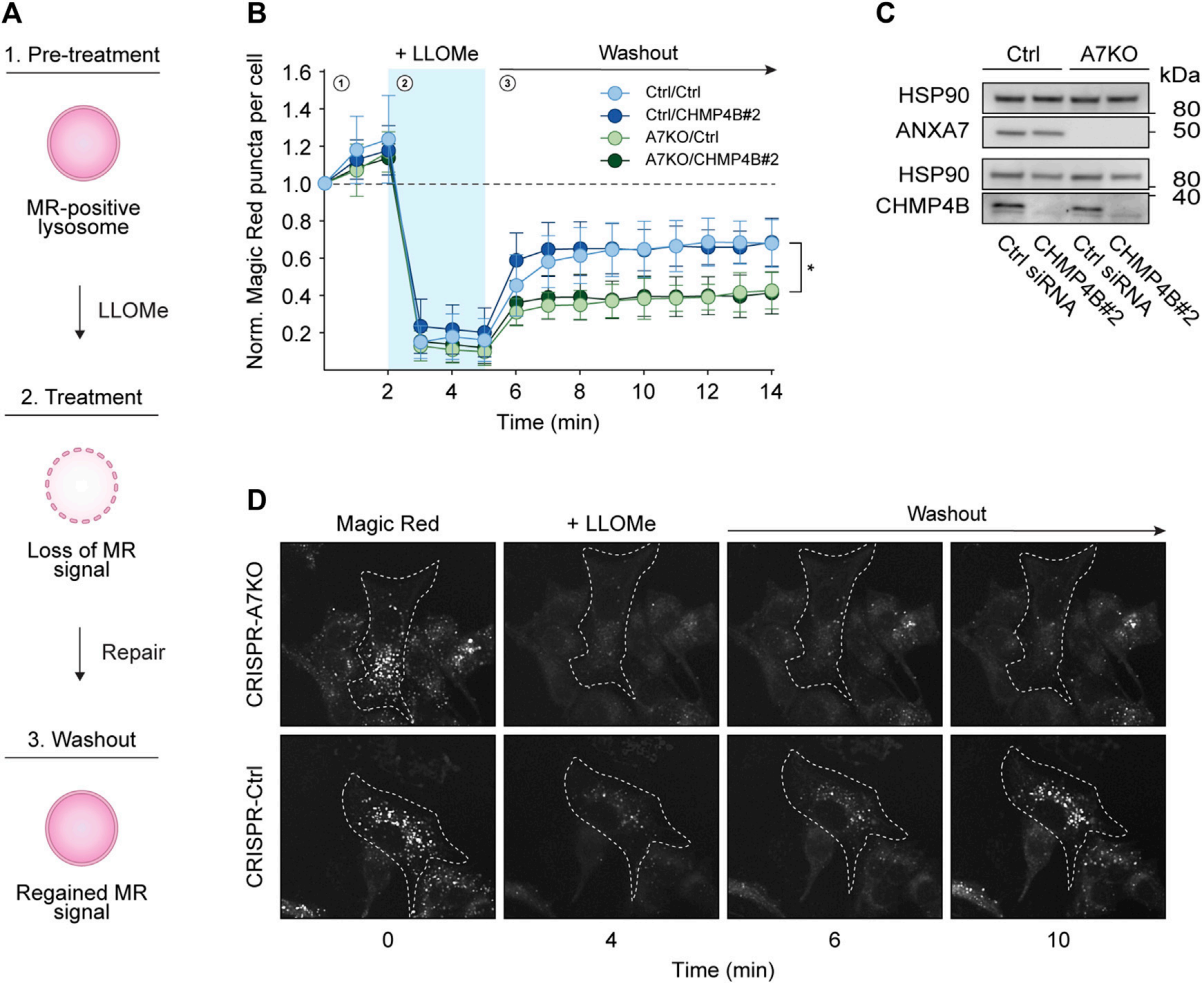
ANXA7 knockout impairs lysosome repair in MCF7-p95ErbB2 cells. **(A)** Schematic representation of the Magic Red (MR) assay. 1) Magic red-positive lysosomes generate a fluorescent signal, which is lost upon 2) LLOMe treatment. 3) The signal intensity is regained concurrently with lysosomal membrane repair, providing a quantifiable readout for repair kinetics. **(B)** Normalized Magic Red puncta per cell over time during 1) pretreatment, 2) LLOMe treatment (1.5 mM) and 3) washout. Data from four independent experiments are quantified from ≥17 cells per condition and depicted as the mean ± SD. The *p*-value was defined by an unpaired two-tailed *t*-test with Welch’s correction comparing the area under the curve from the last time point of LLOMe treatment (5 min) until the endpoint (14 min) between MCF7-p95ErbB2 CRISPR-A7KO and CRISPR-Ctrl cells in each siRNA conditions. *p ≤ 0.05. **(C)** Immunoblot of lysates from MCF7-p95ErbB2 CRISPR-A7KO and CRISPR-Ctrl cells transfected with Ctrl or CHMP4B#2 siRNA showing bands detected with antibodies against ANXA7, CHMP4B and HSP90. Original unedited blots are presented in Supplementary Figure S6. **(D)** Time-lapse images of Magic Red-labeled MCF7-p95ErbB2 CRISPR-A7KO and CRISPR-Ctrl cells transfected with Ctrl siRNA and pulse-treated with LLOMe (1.5 mM). The image intensities have been adjusted for improved visual presentation. Abbreviations: (MR) Magic Red. Remaining abbreviations as in Figures 1, 2.

To more directly assess the putative role of ANXA7 in lysosome repair, we performed the well-established Magic Red lysosome repair assay (Bright et al., 2016; Skowyra et al., 2018; Yim et al., 2022). In this assay, our CRISPR/Cas9 modified MCF7-p95ErbB2 cells were incubated with Magic Red, a chemical substrate that releases a photostable red fluorophore, cresyl violet, upon catalytic cleavage by lysosomal proteases. Loss of the lysosomal pH gradient upon LLOMe treatment results in reduced enzymatic activity, detectable as a loss of fluorescent signal, which is regained upon lysosome repair and restored pH gradient (Figure 4A), hence providing a quantifiable readout for repair kinetics. Once again, we used siRNA depletion of CHMP4B as a benchmark for impaired ESCRT-III-mediated lysosome repair. However, surprisingly we detected no difference in the reoccurrence of Magic Red puncta upon CHMP4B depletion in either CRISPR-A7KO or CRISPR-Ctrl cells despite efficient knockdown (Figures 4B–D; Supplementary Figures S6A–D). By contrast, knockout of ANXA7 significantly impaired the reoccurrence of Magic Red puncta independent of the siRNA condition (Ctrl or CHMP4B#2 siRNA) (Figures 4B, D). Consequently, the data suggest that ANXA7 is more crucial than the ESCRT-III complex for lysosome repair in MCF7-p95ErbB2 cells under the investigated conditions.

### Annexin A7 functions independently from lysophagy

**FIGURE 5:**
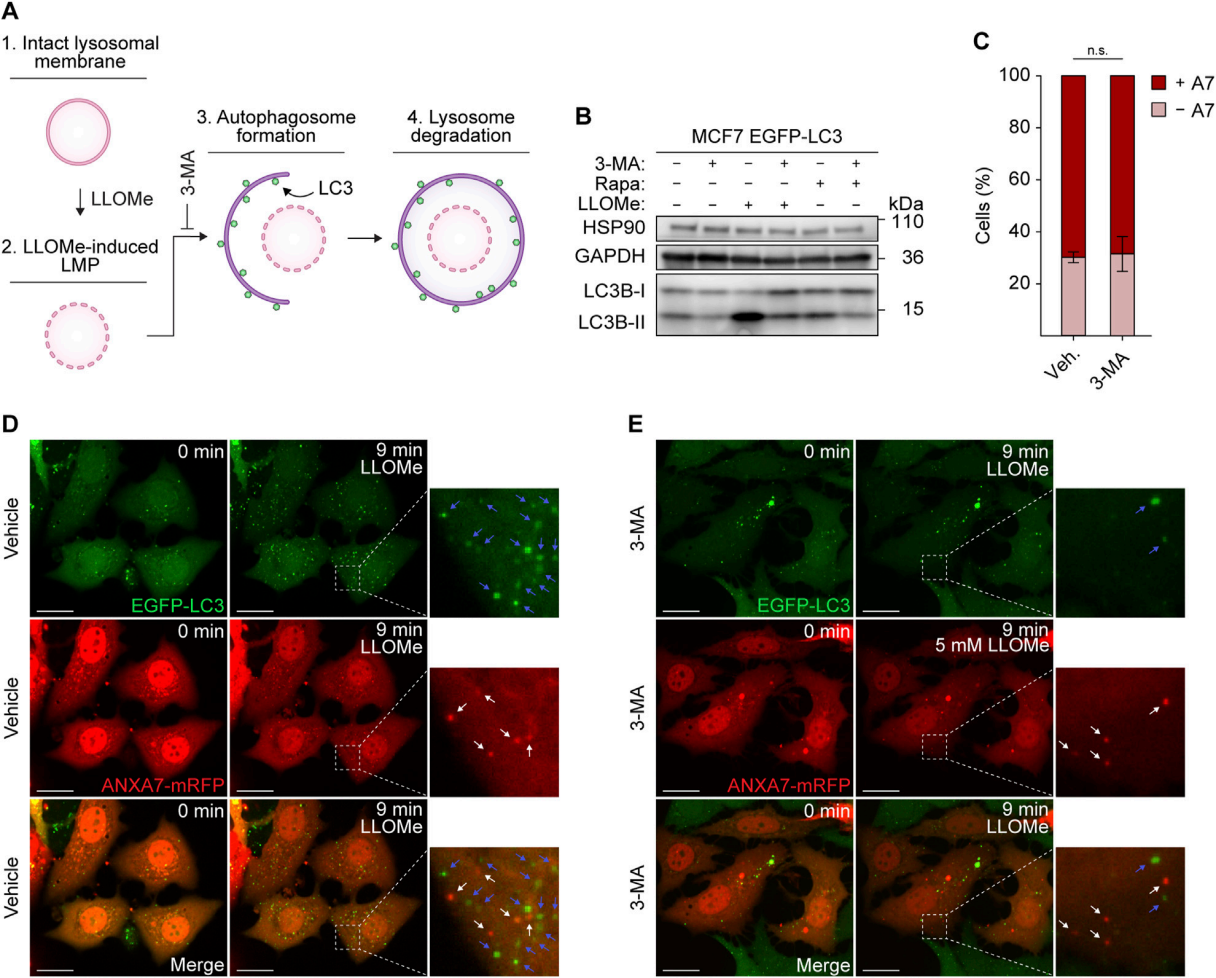
ANXA7 functions separately from lysophagy. **(A)** Schematic representation of lysophagy and relevant factors. 1) The lysosomal membrane undergoes permeabilization upon 2) LLOMe treatment. 3) Maturing autophagosomes, coated with LC3, 4) engulf and degrade damaged lysosomes that are incapable of repair. 3-MA blocking autophagosome formation and the ensuing autophagic steps. **(B)** Immunoblot of lysates from MCF7 EGFP-LC3 cells treated with vehicle (cell culture medium), 3-MA (10 mM, 30 min), LLOMe (5 mM, 15 min) or rapamycin (200 nM, 2 h) showing bands detected with antibodies against HSP90, GAPDH and LC3. Original unedited blots are presented in Supplementary Figure S7. **(C)** Percentage of ANXA7-positive or -negative MCF7 EGFP-LC3 cells in response to LLOMe (5 mM) and pretreatment with 3-MA (10 mM, 30 min) or vehicle (cell culture medium). Cells were quantified as positive if displaying 5 ≥ newly formed ANXA7 puncta within 15 min of LLOMe treatment. Data from three independent experiments are quantified from ≥13 cells per condition and depicted as mean ± SD. The *p*-value was defined by an unpaired two-tailed *t*-test with Welch’s correction. **(D,E)** Representative images of ANXA7 and LC3 puncta formation upon exposure to LLOMe (5 mM) in MCF7 EGFP-LC3 cells transiently co-expressing ANXA7-mRFP. Cells were pretreated with 3-MA (10 mM, 30 min) or vehicle (cell culture medium) prior to exposure to LLOMe. White (ANXA7) and blue (LC3) arrows show puncta in zoomed-in images. Scale bar denotes 20 µm. The image intensities have been adjusted for improved visual presentation. Supplementary Movies S4–S9 of the image sequences is presented in the Supplementary Material. Abbreviations: (3-MA) 3-methyladenine, (GAPDH) glyceraldehyde-3-phosphate dehydrogenase, (LC3) microtubule-associated protein 1A/1B-light chain 3. Remaining abbreviations as in Figure 1.

Recruitment and ESCRT-III-mediated lysosome repair have been observed to precede and function independently of lysophagy (Radulovic et al., 2018; Skowyra et al., 2018). To establish whether ANXA7-mediated lysosome repair also functions separately from lysophagy, we investigated the formation of LLOMe-induced ANXA7 puncta after inhibiting lysophagy using the generic autophagy inhibitor 3-methyladenine (3-MA) (Figure 5A). By suppressing the production of phosphatidylinositol-3-phosphate (PtdIns3P), 3-MA effectively hinders autophagosome formation (Seglen and Gordon, 1982; Blommaart et al., 1997; Høyer-Hansen et al., 2007).

To accomplish this, we used MCF7 cells stably expressing enhanced green fluorescent protein fused to LC3B (referred to as LC3) (MCF7 EGFP-LC3), a widely used marker of autophagosomes (Høyer-Hansen et al., 2007). MCF7 EGFP-LC3 cells co-expressing ANXA7-mRFP and pretreated with 3-MA or vehicle (30 min) were exposed to LLOMe and imaged for 15 min by time-lapse confocal microscopy. Treatment with LLOMe induced profound LC3 lipidation, surpassing the effect observed with the potent autophagy inducer rapamycin (Noda and Ohsumi, 1998), clearly indicating extensive induction of autophagy within the treatment window (Figure 5B). Pretreatment with 3-MA robustly suppressed LC3 lipidation in response to both LLOMe and rapamycin, demonstrating the efficacy of 3-MA in inhibiting autophagy (Figure 5B; Supplementary Figure S7).

As previously observed, cells showed strong ANXA7 puncta formation upon LLOMe treatment, and inhibiting autophagy did not significantly affect the average number of ANXA7-positive cells (Figures 5C–E), showing that ANXA7-mediated lysosome repair, like the ESCRT machinery, operates separately from lysophagy. Further supporting this conclusion, ANXA7 and LC3 puncta did not appear to co-occur (Figures 5D, E).

### ANXA7 is not required for ER-to-lysosome lipid transfer following lysosome damage

In the newly identified PITT pathway, rapid generation of PtdIns4P by phosphatidylinositol-4 kinase type 2α (PI4K2A) at damaged lysosomes facilitates the recruitment of oxysterol-binding protein-related proteins (ORPs), which in turn interact with ER-anchored vesicle-associated membrane protein-associated proteins (VAPs) to orchestrate extensive MCSs between the ER and lysosomes (Radulovic et al., 2022; Tan and Finkel, 2022). Robust ER-to-lysosome transfer of PS and cholesterol through established MCSs promotes subsequent lysosome repair. Interestingly, different members of the ANXA family are becoming recognized for their implication in MCS formation (Enrich et al., 2021). The putative function of ANXAs in MCS formation is hypothesized to originate from FFAT-like motifs (two phenylalanines in an acidic tract) within the ANXAs, which amongst others, would permit interactions with ER-localized VAPs. Phospho-FFAT motifs have been identified in ANXA5 and ANXA8 (Di Mattia et al., 2020; Enrich et al., 2021), while homologies to FFAT motifs have been identified in ANXA1, ANXA6, and ANXA11 (Rentero et al., 2018; Enrich et al., 2021). Accordingly, ANXA6 has been implicated in MCS formation and ER-to-late endosome/lysosome transfer of cholesterol (Meneses-Salas et al., 2020). However, given the extensive interaction between ANXAs and ANXA binding partners, their implication in MCS formation should not be restricted solely to family members containing phospho- or FFAT-like motifs. Supportive hereof, ANXA7 and ANXA11 were identified as binding partners when using ANXA6 as a bait (Enrich et al., 2021).

**FIGURE 6:**
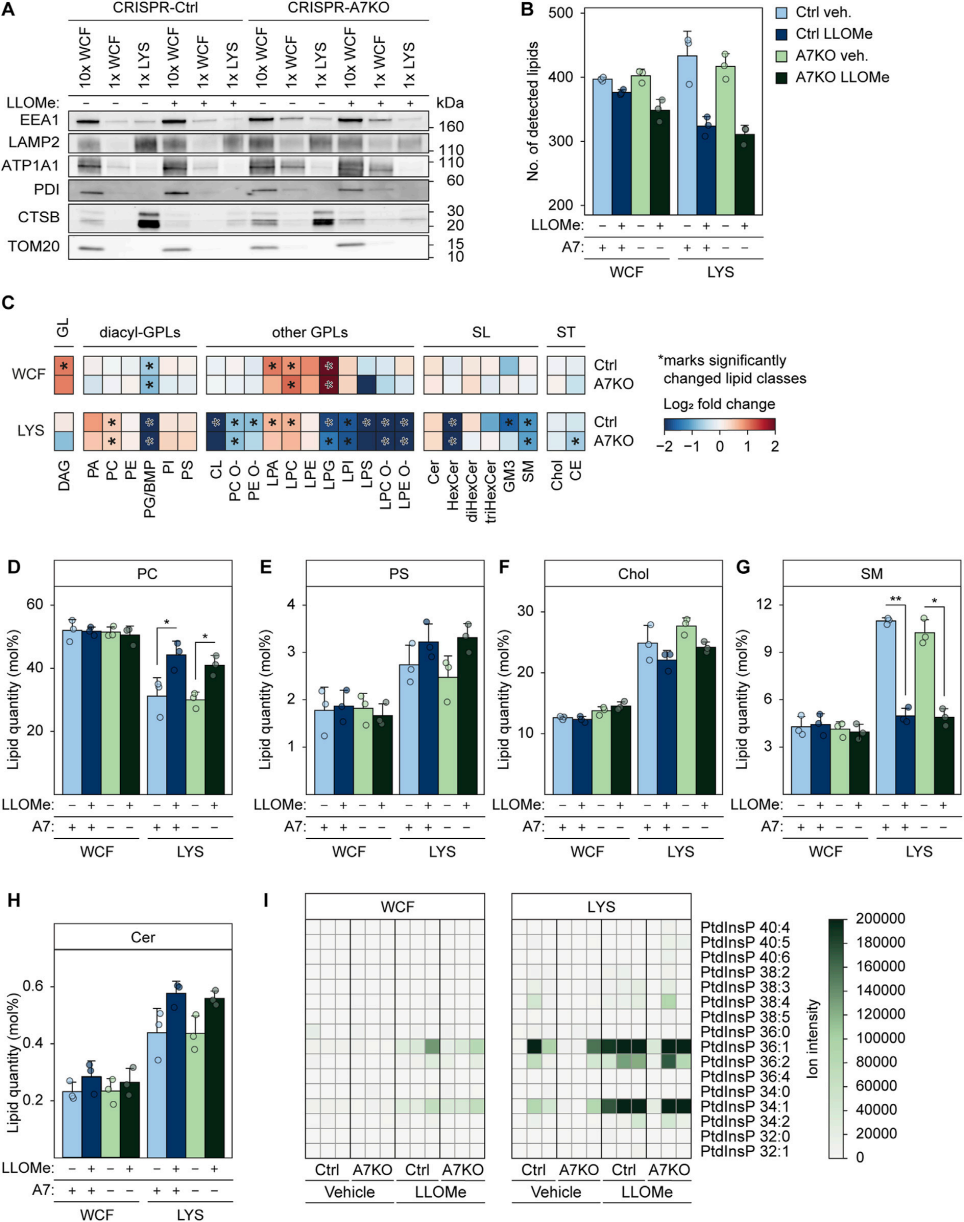
Damage-induced alterations in lysosome lipid composition are unaffected by ANXA7 knockout. Immunoblot and lipidomics analysis of WCF or purified LYS from MCF7-p95ErbB2 CRISPR-A7KO and CRISPR-Ctrl cells treated with vehicle (diH_2_O) or LLOMe (2 mM) for 1 h. **(A)** Immunoblot of WCF and LYS fractions with protein markers of the indicated cellular compartments. Loading was adjusted according to the total lipid content. Original unedited blots are presented in Supplementary Figure S8C. **(B)** Total number of detected lipid species according to treatment. Total molar quantity of lipids identified according to treatment are presented in Supplementary Figure S8A. **(C)** Heatmaps showing log_2_ fold change of individual lipid classes in LLOMe-vs. vehicle (diH_2_O)-treated cells. Significantly changed lipid classes (*q*-value ≤0.05) are marked with an asterisk regardless of the magnitude of the *q*-value. **(D)** Levels of PC given in mol%. **(E)** Levels of PS given in mol%. **(F)** Levels of cholesterol given in mol%. **(G)** Levels of SM given in mol%. **(H)** Levels of ceramide given in mol%. Circles represent the individual replicates in **(B)**, **(D–H)**. Linear modeling with a Benjamini-Hochberg correction was used to determine statistical significance in **(B)**, **(D–H)**. *p ≤ 0.05; **p ≤ 0.01. **(I)** Ion intensities of PtdInsPs detected in the MS1 spectra. Values from each replicate are depicted in separate rows. Ion intensities of PtdInsPs detected in the MS2 spectra are presented in Supplementary Figure S6B. Abbreviations: (ATP1A1) sodium/potassium-transporting ATPase subunit alpha-1, (CE) cholesteryl ester, (Cer) ceramide, (Chol) cholesterol, (CL) cardiolipin, (DAG) diacylglycerol, (diHexCer) dihexosylceramide, (GM3) ganglioside GM3, (HexCer) hexosylceramide, (PA) phosphatidic acid, (LPA) lysoPA, (PC) phosphatidylcholine, (PC O-) alkyl-PC, (LPC) lysoPC, (LPC O-) alkyl-LPC, (PE) phosphatidylethanolamine, (PE O-) alkyl-PE, (LPE) lysoPE, (LPE O-) alkyl-LPE, (PG) phosphatidylglycerol, (LPG) lysoPG, (PI) phosphatidylinositol, (LPI) lysoPI, (PS) phosphatidylserine, (LPS) lysoPS, (PtdInsP) phosphatidylinositol phosphate, (SM) sphingomyelin, (triHexCer) trihexosylceramide. Remaining abbreviations as in Figure 1.

In light of this, we wondered whether ANXA7 might be part of orchestrating the formation of ER-lysosomal MCSs to facilitate PITT-mediated lysosome repair. As for the PITT pathway, we demonstrate that ANXA7 mediates lysosome repair independently of ESCRT-III (Figures 3A, B), highlighting a novel repair role of ANXA7 differing from its known function in ESCRT-III-mediated plasma membrane repair (Sønder et al., 2019). We approached the question by performing quantitative mass spectrometry (MS)-based shotgun lipidomic analysis of FeDEX-purified lysosomes from MCF7-p95ErbB2 CRISPR-A7KO and CRISPR-Ctrl cells treated with LLOMe or vehicle (diH_2_O) (1 h). We identified around 300 or more different lipid species belonging to 27 lipid classes distributed in the categories glycerolipids (GLs), glycerophospholipids (GPLs), sphingolipids (SLs), and sterol lipids (Figure 6B; Supplementary Figure S8A). We absolutely quantified them and expressed their quantities in molar percentages (mol%) of all identified lipid species to determine the stoichiometry between lipids in the samples.

The FeDEX-based isolation procedure once again successfully enriched lysosomal markers, LAMP2 and CTSB, whereas cellular markers for early endosomes (EEA1), plasma membrane (ATP1A1), ER (PDI) and mitochondria (TOM20) were vastly depleted (Figure 6A; Supplementary Figure S8D). Moreover, the lysosomal fractions obtained from vehicle-treated cells were abundant in cholesterol, sphingomyelin, and the endolysosome-specific lipid bis (monoacylglycero) phosphate (BMP) (Figures 6F,G; Supplementary Figure S8C) (Möbius et al., 2003; Tharkeshwar et al., 2017), in agreement with previously reported lipid profiles of lysosomes obtained from various mammalian cells (van Meer et al., 2008; Bilgin et al., 2017; Tharkeshwar et al., 2017; Nielsen I. et al., 2020; Scrima et al., 2022; Stahl-Meyer et al., 2022).

LLOMe treatment did not significantly alter the mol% values of most lipid classes in the whole-cell fraction in either of the two cell lines (Figure 6C; Supplementary Figure S8C); exceptions include BMP and certain lysoglycerophospholipid (lysoGPL) classes, which decreased (BMP) or increased (lysoGPL) significantly upon LLOMe treatment in line with previously reported effects of other LMP-inducing drugs (Nielsen I. et al., 2020; Fogde et al., 2022). In contrast, LLOMe treatment significantly altered several lipid classes in the lysosomal fractions (Figure 6C). In agreement with damaged lysosomes initiating the PITT pathway by forming MCSs to the PC-rich ER and subsequently transferring ER-synthesized PS (Radulovic et al., 2022), the LLOMe treatment increased PC and PS in the obtained lysosomal fractions (Figures 6C–E). Furthermore, even though not quantifiable due to the lack of a standard in our lipidomics pipeline, we detected ions matching multiple PtdIns4P species almost exclusively after LLOMe treatment, particularly prominently in the lysosomal fractions (Figure 6I; Supplementary Figure S8B). We observed a non-significant reduction of cholesterol upon LLOMe treatment in the lysosomal fraction (Figure 6F), even though the ER-to-lysosome transfer of cholesterol was previously proposed as a crucial event in the PITT pathway (Radulovic et al., 2022; Tan and Finkel, 2022). The expected increase in lysosomal cholesterol was possibly masked by the large increase in the highly abundant PC (Figure 6D). Notably, LLOMe treatment also triggered a significant reduction in sphingomyelin and an increase in ceramide in the isolated lysosomes (Figures 6C, G, H), indicating that sphingomyelin was hydrolyzed at the surface of damaged lysosomes to form ceramide, as previously proposed as a mechanism of lysosome repair (Niekamp et al., 2022). All the above-described lipid changes induced by the LLOMe treatment occurred to a similar extent in lysosomes isolated from MCF7-p95ErbB2 CRISPR-A7KO and CRISPR-Ctrl cells (Figures 6C–I; Supplementary Figures S8A–C). In summary, our lipidomics data strongly suggest that MCF7-p95ErbB2 cells can promote lysosome repair through the newly identified PITT pathway (Radulovic et al., 2022; Tan and Finkel, 2022), while also revealing that ANXA7 is expendable for the formation of ER-lysosomal MCSs and subsequent lipid transfer.

## Discussion

In this study, we show that endogenous ANXA7 and ALG-2 co-purifies with lysosomal markers upon LLOMe treatment, suggesting that ANXA7 and ALG-2 are recruited to damaged lysosomes (Figures 1G, H; Supplementary Figure S1). In support hereof, fluorescently-tagged ANXA7 translocates to GAL3-positive vesicles following LLOMe treatment (Figure 2E; Supplementary Movie S3), whereas no puncta formation or translocation was observed for fluorescently-tagged ANXA2 and–ANXA5 (Figures 2F, G; Supplementary Movies S4, S5), indicating specificity of ANXA7 towards damaged lysosomes in MCF7 cells as compared to ANXA2 and ANXA5. We show that the translocation of ANXA7 to damaged lysosomes in MCF7 cells was independent of ALG-2 (Figures 2A, B; Supplementary Movies S1, S2), which is in line with our previous demonstration of ANXA7’s requirement in plasma membrane repair through the recruitment of ESCRT-III components (Sønder et al., 2019). In this repair pathway, ALIX functions as a downstream effector of ANXA7 and ALG-2. Hence, the recruiting role of ALIX in cooperation with TSG101 during ESCRT-III-mediated lysosome repair naturally questioned a potential mechanistic overlap (Skowyra et al., 2018; Radulovic et al., 2022). These results prompted us to investigate whether ANXA7 likewise promotes ESCRT-III-mediated lysosome repair. Surprisingly, ANXA7 knockout did not impair CHMP4B buildup and ESCRT-III assembly upon lysosome damage (Figures 3A–C), demonstrating that different mechanisms exist for ESCRT-III recruitment at the plasma membrane and lysosomes. In line with this, Radulovic et al. (2018) reported that depletion of ALIX alone did not affect CHMP4B buildup, whereas depletion of TGS101 by itself had a moderate effect, suggesting that TSG101 plays a larger role than ALIX in ESCRT-III recruitment to damaged lysosomes. CHMP4B buildup was only completely abolished upon simultaneous depletion of ALIX and TSG101 (Radulovic et al., 2018), suggesting that ALIX and TSG101 act cooperatively in recruiting ESCRT-III. Thus, we cannot exclude the possibility that ANXA7/ALG-2 may recruit ALIX to damaged lysosomes, where ALIX in cooperation with TSG101 would facilitate ESCRT-III assembly, since only obstructing the function of ALIX would not affect CHMP4B buildup (Radulovic et al., 2018). Nevertheless, ESCRT-III recruitment at the plasma membrane and lysosomes would still be mechanistically distinct as ANXA7 knockout completely abolishes CHMP4B buildup at the plasma membrane (Sønder et al., 2019), suggesting a more prominent role of ANXA7 in ESCRT-III-mediated plasma membrane repair.

By contrast, knockout of ANXA7 significantly increased GAL3 accumulation, indicating an increase in damaged lysosomes in response to LLOMe treatment compared to control cells (Figures 3C–E). This increase in GAL3 accumulation occurred independently of ESCRT-III recruitment (Supplementary Figures S4A–C), consistent with previous demonstrations (Radulovic et al., 2018; Skowyra et al., 2018). We hypothesize that since ESCRT-III-mediated repair is presumably facilitated at the cytoplasmic surface of lysosomes, the complex will have access to subtle membrane lesions not yet large enough to grant access to intralumenal β -galactosides. In support of this, ANXA1 and ANXA2 were recently shown to primarily translocate to and facilitate the repair of larger lysosomal lesions (>4.6 nm) (Yim et al., 2022), likely aiding ESCRT-III-mediated repair. Notably, Yim et al. (2022) reported that all investigated ANXAs (ANXA1, A2, A4, A5, A6, A7, and A11) localized at damaged lysosomes in U2OS cells, but only ANXA1 and ANXA2 promoted lysosome repair. Contrarily, our data demonstrate that ANXA7 promotes lysosome repair in invasive MCF7-p95ErbB2 cells (Figures 4B, D). Importantly, our findings suggest that this repair process operates independently of lysophagy (Figures 5B–E; Supplementary Movies S6, S7) aligning with observations from previous studies (Radulovic et al., 2018; Skowyra et al., 2018). The existing data suggest a cell type specificity of ANXAs for damaged lysosomes in agreement with our lysosomal fractionation data, showing that ANXA1 and ANXA5 co-purifies with lysosomal markers in addition to ANXA7 and ALG-2 upon LLOMe treatment in HeLa cells (Figure 1H; Supplementary Figure S1). Collectively, these data contest the concept of ANXA7/ALG-2 facilitating ESCRT-III recruitment to damaged lysosomes via ALIX.

It seems conceivable that the distinct morphologies of ANXA-induced membrane curvature may aid different types or phases of the repair process (Boye et al., 2018). For instance, in the recently identified sphingolipid-operated lysosome repair pathway, calcium-activated sphingomyelin scrambling and turnover create ceramide microdomains at the cytosolic surface of damaged lysosomes, hypothesized to mediate ESCRT-III-independent repair through the induction of negative membrane curvature (Niekamp et al., 2022). Interestingly, ceramide increases the calcium sensitivity of ANXA1-membrane interactions (Babiychuk et al., 2008), indicating a potential synergistic effect between ANXAs and ceramide microdomains at the cytosolic surface of damaged lysosomes, especially when considering the folding and blebbing morphology displayed by ANXA1 and ANXA2 at artificial open-edge membranes (Boye et al., 2018). In support, ANXA2 is reported to induce negative membrane curvature in a calcium-dependent manner sufficient to generate vesicles from parental giant vesicles (Drücker et al., 2013; Hakobyan et al., 2017; Boye et al., 2018).

ANXAs are becoming increasingly recognized for their multiple roles in MCS formation and functioning (Enrich et al., 2021), which are believed to be mediated via FFAT-like motifs within certain ANXA members (ANXA1, A5, A6, A8, and A11) (Rentero et al., 2018; Di Mattia et al., 2020; Enrich et al., 2021). These motifs would, amongst others, permit interactions with ER-localized VAPs, the latter required for establishing contact between the ER and damaged lysosomes (Radulovic et al., 2022). Since the role of ANXA7 in lysosome repair differs from that at the plasma membrane, we hypothezied that ANXA7 might contribute to MCS formation in the newly identified PITT pathway (Radulovic et al., 2022; Tan and Finkel, 2022). However, given the lack of FFAT-like motifs within ANXA7, such a potential role would have to be mediated in cooperation with ANXA7 interaction partners. We indirectly investigated the putative function of ANXA7 in orchestrating ER-lysosomal MCSs by quantitative MS-based shotgun lipidomics analysis of FeDEX-purified lysosomes from MCF7-p95ErbB2 CRISPR-A7KO and CRISPR-Ctrl cells treated with LLOMe. These data revealed an increase in PC, PS and PtdIns4P in the lysosomal fractions after LLOMe treatment (Figures 6C–E, I; Supplementary Figure S8B) to an extent similar to findings for the PITT pathway (Radulovic et al., 2022), strongly suggesting MCF7-p95ErbB2 cells to facilitate lysosome repair through this pathway. Despite detecting a paradoxical reduction in cholesterol upon treatment, which is inconsistent with recent findings (Figure 6F) (Radulovic et al., 2022; Tan and Finkel, 2022), we speculate that the expected increase in lysosomal cholesterol may have been masked by the significant increase in the highly abundant PC (Figure 6D). Moreover, differences in both the experimental timeline and cholesterol metabolism among cell lines would also likely impact the outcome of ER-to-lysosome lipid transfer (Radulovic et al., 2022; Tan and Finkel, 2022).

Furthermore, we observed that LLOMe triggered a significant reduction in sphingomyelin and an increase in ceramide in isolated lysosomes (Figures 6G, H), consistent with recent findings indicating that calcium-activated sphingomyelin scrambling and turnover can mediate lysosome repair by creating ceramide microdomains at the cytosolic surface of damaged lysosomes (Niekamp et al., 2022). Here, we provide supportive evidence for this repair mechanism by demonstrating the reported findings as changes in endogenous lipid content. However, it should be noted that the conversion of sphingomyelin to ceramide cannot solely account for the significant reduction in sphingomyelin observed upon LLOMe treatment (Figure 6G).

Even though the lipidomics data support the existence of two novel lysosome repair pathways, we detected no significant differences of biological importance in the lysosomal fraction between MCF7-p95ErbB2 CRISPR-A7KO and CRISPR-Ctrl cells after LLOMe treatment (Figures 6C–I; Supplementary Figures S8B, C). Therefore, it appears ANXA7 is not necessary for orchestrating the formation of ER-lysosomal MCSs following lysosome damage. It could be tempting to speculate that the unaltered lipid profile upon ANXA7 knockout may be due to compensatory actions from other ANXA family members in the ANXA7KO background. We cannot dismiss this possibility, as we have not investigated potential alterations in expression levels of other ANXAs in response to ANXA7 knockout. However, collectively our data clearly demonstrate a specific role of ANXA7 in lysosome repair since knockout significantly impaired repair and increased lysosomal GAL3 accumulation (Figures 3D–E, 4B, D; Supplementary Figures S4A, B). Based on this, it would be sensible if ANXA7 knockout had enriched ER-associated lipid classes in the lysosomal fraction upon damage to compensate for the lack of ANXA7-mediated lysosome repair. However, this is not the case (Figures 6C–I; Supplementary Figures S8B, C), and we speculate whether this could be attributable to ANXA7 and the PITT pathway acting at different independent phases of the collective repair process. The understanding that the need for acute repair to restore membrane permeability is followed by an equally important need for membrane restructuring to regain functionality has gained growing appreciation in later years. The primary distinguishing factor between these repair phases is the timescale at which they occur, as acute repair typically occurs within seconds (Andrews et al., 2014; Cooper and McNeil, 2015; Häger and Nylandsted, 2019; Dias and Nylandsted, 2021; Klenow et al., 2021), whereas subsequent membrane restructuring occurs minutes to hours post-injury (Cooper and McNeil, 2015; Häger and Nylandsted, 2019; Dias and Nylandsted, 2021; Sønder et al., 2021). Notably, Radulovic et al. (2022) reported an increment in ER-associated lipid classes in the lysosomal fraction from 10 to 45 min post-injury. The extensive timescale of PITT-mediated lysosome repair indicates that ER-to-lysosome lipid transfer likely supports membrane restructuring to reestablish lysosome function. In contrast, ANXA1, ANXA2, ANXA7 and the ESCRT machinery facilitate lysosome repair at a considerably shorter timescale (Figures 4B, D) (Radulovic et al., 2018; Skowyra et al., 2018; Yim et al., 2022), more in line with expectations for acute repair. A difference in the function of these repair pathways could potentially explain why the PITT pathway can be activated and function independently of not only ANXA7 but also the ESCRT machinery (Radulovic et al., 2022), although this remains to be investigated in greater detail.

**FIGURE 7:**
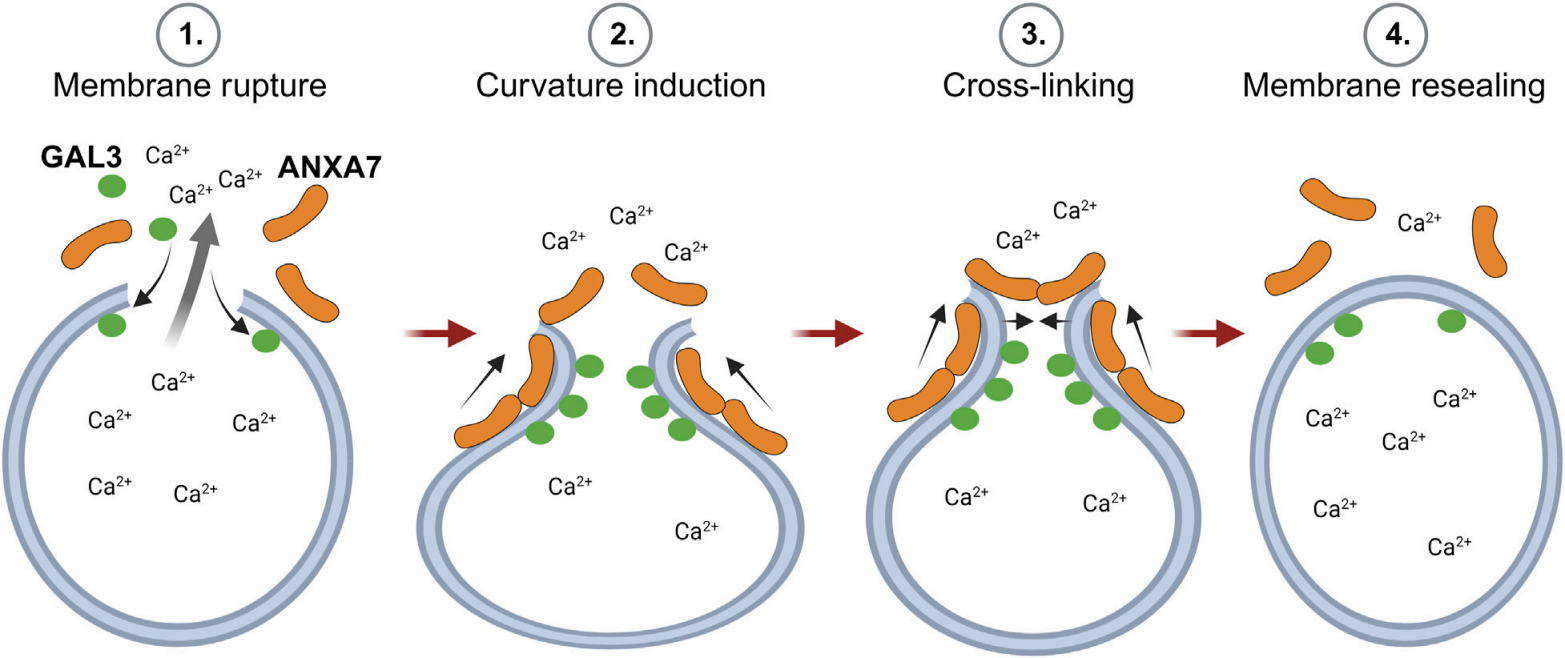
Proposed model for ANXA7-mediated lysosome repair through membrane curvature, cross-linking and fusion. 1) Lysosome damage leading to compromised membrane integrity results in the spillage of lysosomal content and severely threatens cell viability. 2) Larger lysosomal lesions permit the binding of damage marker, GAL3, to exposed intralumenal β-galactosides and trigger calcium (Ca^2+^)-dependent recruitment of ANXA7 to the injury site. Here, ANXA7 likely facilitates repair through induction of membrane curvature, cross-linking and membrane fusion. The Figure is created with BioRender.com. Abbreviations as in Figure 1.

In previous studies, we demonstrate that ANXA7 induces local curvature at open-edge artificial membranes (Boye et al., 2018; Sønder et al., 2019). This, in conjunction with ANXA7’s established capacity for self-association at membranes to induce membrane fusion and cross-linking (Creutz et al., 1979), along with the findings presented in this study, indicate that ANXA7 likely facilitates lysosome repair through induction of membrane curvature, cross-linking and membrane fusion (Figure 7). In support, a mechanism depending on classic ANXA functions, rather than the unique characteristics of a specific family member, could potentially explain the redundancy of ANXAs in lysosome repair (Yim et al., 2022).

Overall, our results suggest that Annexin A7 mediates lysosome repair independently of ESCRT-III. Our data furthermore support newly identified mechanisms of lysosome repair and contribute to the general appreciation of lysosome repair as a dynamic and multi-layered process with different independent mechanisms complimenting each other (Radulovic et al., 2018; Skowyra et al., 2018; Niekamp et al., 2022; Radulovic et al., 2022; Tan and Finkel, 2022; Yim et al., 2022). We expect the current knowledge base and understanding of the complementary interplay between these and novel lysosome repair pathways will expand in the coming years, ultimately highlighting the importance of lysosomal integrity in cellular health.

## Materials and methods

### Cell culturing and drug treatments

Human breast adenocarcinoma MCF7 and human cervix adenocarcinoma HeLa cells were cultured in Roswell Park Memorial Institute (RPMI) 1640 medium containing GlutaMAX (Termo Fisher Scientific, 61870) and supplemented with 6% (*v/v*) heat-inactivated fetal bovine serum (FBS) (Termo Fisher Scientific, 10270-106) and 0.25% (*v/v*) penicillin-streptomycin (Life Technologies, 11811-031). Cells were kept at 37°C in a humidified atmosphere with 5% CO_2_ as a sub-confluent monolayer and were routinely tested for the presence of *mycoplasma* (Minerva Biolabs Inc., Venor®GeM Classic). The MCF7 S1 clone and its derivatives expressing EGFP-LC3B or EGFP-LGALS3 have been previously described (Jäättelä et al., 1995; Høyer-Hansen et al., 2007; Aits et al., 2015). MCF7-p95ErbB2 cells are single cell clones of the MCF7 S8 cell line stably expressing a truncated version of ErbB2 (p95ErbB2) under the transcriptional control of a tetracycline-controlled transactivator (Egeblad et al., 2001). These clones were further modified using CRISPR/Cas9 technology to generate an ANXA7 knockout (CRISPR-A7KO) and control (CRISPR-Ctrl) cell line (Sønder et al., 2019). MCF7-p95ErbB2 cells were cultivated in RPMI 1640 medium supplemented with 1 μg/mL tetracycline (Sigma-Aldrich, T7660), G418 Sulfate (Life Technologies, 11811-031) and 1 μg/mL puromycin (Sigma-Aldrich, P7255) in addition to FBS and penicillin-streptomycin. All experiments were performed 3–5 passages after p95ErbB2 induction by removing tetracycline from the cell culture medium.

Live-cell imaging was performed in cell-imaging medium (CIM) consisting of RPMI 1640 medium without phenol red (Termo Fisher Scientific, 32404014) supplemented with 2 mM GlutaMAX (Thermo Fisher Scientific, 35050038), 6% (*v/v*) FBS, 0.25% (*v/v*) penicillin-streptomycin and 25 mM HEPES (Sigma-Aldrich, H3375).

FeDEX-mediated lysosomal isolation was performed in Dulbecco’s modified Eagle’s medium (DMEM) containing GlutaMAX (Termo Fisher Scientific, 31966021) supplemented with 6% (*v/v*) FBS and 0.25% (*v/v*) penicillin-streptomycin to reduce FeDEX precipitation.

LMP was induced using LLOMe (Santa Cruz Biotechnology, SC-285992) dissolved in autoclaved deionized water (diH_2_O). The applied concentration and incubation period are specified in the figure legends of the individual experiments. Autophagy was suppressed by exposing cells to 10 mM 3-MA (Sigma-Aldrich, M9281) dissolved in CIM for 30 min, whereas a 2 h treatment with 200 nM rapamycin (Sigma-Aldrich, 553210) dissolved in DMSO served as a positive control of autophagy induction.

### FeDEX-mediated lysosomal isolation

Lysosomes were isolated using FeDEX particles (40 kDa) (Bilgin et al., 2017). FeDEX was prepared in solution as previously described (Bilgin et al., 2017) and no more than a week before experiments. Lysosomes were loaded with FeDEX using a Pulse-Chase approach, where cells were incubated overnight (ON) in cell culture medium containing 0.66% (*v/v*) FeDEX solution followed by a 3-h chase in clean medium. Vehicle or 2 mM LLOMe was added to the cell culture medium during the last hour of the chase period. Cells were harvested by scraping, spun down at 500 × g for 2 min at 4°C and washed in ice-cold Dulbecco’s phosphate-buffered saline (DPBS) (Thermo Fisher Scientific, 14190094). Cells were subsequently lysed mechanically in a hypotonic SCA buffer (10 mM HEPES, 210 mM mannitol, 70 mM sucrose, pH 7.5) supplemented with 1 M DTT, ×1 Complete mini EDTA-free protease inhibitor cocktail (Roche, 4693124001) and 1 µL Benzonase^®^ Nuclease (≥250 units/µL) (Sigma-Aldrich, E1014) (SCA+++ buffer) using a 10 mL syringe and G25 needle (HeLa: ×5 strokes, MCF7: ×15 strokes). The crude cell lysate was spun down at 800 × g for 10 min at 4°C, and the supernatant was transferred to a new Eppendorf tube and spun down at 800 × g for 10 min at 4°C. The cleared supernatant was loaded onto an equilibrated MS column (Miltenyi Biotec, 130-042-201) mounted on a magnetic stand (Miltenyi Biotec, 130-042-109) placed at 4°C, and the column was washed repeatedly with SCA buffer before eluting the lysosomal fraction using a plunger.

### NAG activity assay

Whole-cell and lysosomal fractions were diluted 1:20 in SCA buffer, treated with vehicle (diH_2_O) or 250 μg/mL digitonin (Sigma-Aldrich, D141), and briefly vortexed before being aliquoted to a non-transparent, flat-bottomed 96-well plate (Greiner Bio-One, 655090). Next, 0.3 mg/mL 4-Methylumbelliferyl N-acetyl-β-D-glucosaminide (4-MUF-NAG) (Sigma-Aldrich, M2133) dissolved in 0.2 M citrate buffer (pH 4.5) was added, and the release of fluorogenic 4-Methylumbelliferyl was measured using a Varioskan Flash plate reader (Thermo Fisher Scientific) and SkanIt^®^ Research Edition Software for Varioskan^®^ Flash (v2.4.5). The plate was allowed to acclimate inside the reader for 4 min before measuring the fluorescent signal at 448 nm every 45 s for 20 min at 37°C.

### Western blot analysis

Whole-cell and cellular fractions were lysed in Laemmli sample buffer (125 mM Tris (pH 6.7), 20% (*v/v*) glycerol, 140 mM SDS, 0.3 µM bromophenol blue) supplemented with 0.1 M DTT, cOmplete™, Mini Protease Inhibitor Cocktail (Roche, 4693124001) and PhosSTOP™ (Roche, 4906837001). Lysates were boiled, and proteins were separated by SDS-PAGE using a 4%–15% TGX™ Precast Protein Gel (BioRad, 4561086/5671085) and blotted onto a nitrocellulose membrane (BioRad, 1704158/59) using a Trans-Blot TurboTM transfer system. Novex Sharp Protein Standard (Thermo Fisher Scientific LC5800) was used to evaluate the molecular weights of proteins from the gels. Following blocking in PBS containing 0.1% (*v/v*) Tween-20 (PBST) and 5% (*w/v*) BSA, membranes were cut in appropriate sizes for detection of proteins of interest and incubated ON at 4°C with primary antibodies in 0.1% (*v/v*) NaN_3_/5% (*w/v*) BSA/PBST (Supplementary Table S1). Next, membranes were incubated for 30 min with matching secondary HRP-conjugated antibodies (Supplementary Table S1) and the immunoreactivity was detected using a luminescent image reader (Fujifilm, LAS-4000) following incubation with Clarity™ Western enhanced chemiluminescent substrate (BioRad, 1705061).

### Plasmids and siRNA transfections

Expression plasmids with ANXA2 with a monomeric RFP C-terminal tag and ANXA5 with a turbo RFP C-terminal tag have been constructed by subcloning cDNA sequences of human ANXA2 (OriGene RG205081) or ANXA5 (OriGene RG205619) into the respective mammalian vectors, pCMV6-AC-mRFP (OriGene PS100041) or pCMV6-AC-tRFP (OriGene PS100034) using the restriction enzymes AsiSI and MluI. ANXA7-mRFP was constructed previously using the same approach (Sønder et al., 2019). EGFP-LGALS3 was constructed by subcloning human LGALS3 (isoform 1) with an N-terminal enhanced GFP-tag into the lentiviral CDH-EF1-MCS-IRES-Neo vector as previously described (Aits et al., 2015). Cells were transiently transfected with the described plasmids using Lipofectamine LTX (Invitrogen, 15338100) according to the manufacturer’s instructions, omitting the Plus Reagent, and used for live-cell imaging experiments the day following transfection.

Reverse siRNA transfection was performed using Oligofectamine Transfection Reagent (Invitrogen, 12252011) according to the manufacturer’s instructions. The cell culturing medium was replaced following 24 h, and experiments were performed 72 h after transfection. AllStar Negative Ctrl siRNA (Qiagen, 1027281), ALG-2 [5′-CAC GAC AUC CUC AUU CGA A (dT) (dT)-3′], ANXA7 [5′-GCU UAU UCU AGG AUG GCU A (dT) (dT)-3′], CHMP4B#1 [5′-CGC UAU GCC GCC AAG GCC A (dT) (dT)-3′] and CHMP4B#2 siRNA [5′-CAA GAA CUU GCA GAG GAG A (dT) (dT)-3′] were used at 25 nM.

### Immunocytochemistry

Cells were grown on 170 μm glass coverslips and fixed in 4% (*v/v*) paraformaldehyde (Ampliqon, 43226.1000) for 15 min. Fixed samples were quenched in 50 mM NH_4_Cl for 10 min to reduce the background signal before being permeabilized in ice-cold methanol for 10 min at −20°C. Samples were subsequently blocked for at least 1 h in buffer 1 [1% (*w/v*) BSA, 0.3% (*v/v*) Triton X-100, DPBS] containing 5% (*v/v*) goat serum and incubated for 2 h with primary antibodies in buffer 1. Primary antibodies raised against human CHMP4B (Proteintech, 13683-1-AP, RRID:AB_2877971, 1:200), GAL3 (Sigma-Aldrich, MABT51, RRID:AB_10806498, 1:300) and LAMP2 (Developmental Studies Hybridoma Bank, H4B4, RRID:AB_528129, 1.2 μg/mL) were used. Following incubation with primary antibodies, samples were incubated for 1 h with Goat anti-Rat IgG (H+L) Highly Cross-Adsorbed Secondary Antibody, Alexa Fluor™ Plus 488 (Invitrogen, A48262, RRID:AB_2896330, 1:1,000) and Donkey anti-Mouse IgG (H+L) Highly Cross-Adsorbed Secondary Antibody, Alexa Fluor™ 568 (Invitrogen, A10037, RRID:AB_2534013, 1:1,000) in buffer 2 [0.25% (*w/v*) BSA, 0.1% Triton X-100 (*v/v*), DPBS]. Nuclei were stained with 2.5 μg/mL Hoechst 33342 (Sigma-Aldrich, #B2261) for 10 min in buffer 3 [0.05% (*v/v*) Tween-20, DPBS]. Coverslips were mounted on microscope slides using ProLong™ Gold Antifade Mountant (Invitrogen, P36934).

### Microscopy analysis and live-cell imaging

Images were captured using a LSM 700 confocal microscope (Zeiss) equipped with a Zeiss Plan-Apochromat ×63/1.40 Oil DIC M27 objective and 405 nm, 488 nm, and 555 nm lasers and Zeiss imaging software (v7). CHMP4B and GAL3 puncta were quantified by detecting puncta objects based on fluorescent intensity and pixel size within a defined cell using the CellProfiler analysis software (v4.2.1). Manders’ overlap coefficient was calculated using the JACoP plugin with manual thresholding in the ImageJ software (v2.9.0) (Bolte and Cordelières, 2006; Schneider et al., 2012).

Cells grown in MatTek imaging-culture dish were kept at 37°C in CIM. Live-cell images were captured using an inverted microscope Eclipse Ti-E (Nikon) paired with an UltraVIEW VOX Spinning Disk [PerkinElmer and Volocity software (v6.3.1)] or a Crest X-Light V3 Spinning Disk [CrestOptics and NIS-Elements Advanced Research software (v5.42.03)]. The Eclipse Ti-E was equipped with a Nikon CFI Apochromat ×40 WI λS, NA 1.25, WD 0.18 objective or a Zeiss C Plan-Apochromat ×63/1.2 Water Korr objective and 488 nm and 555 nm lasers. MCF7 EGFP-LGALS3 cells transiently transfected with ANXA2-mRFP or ANXA5-tRFP were imaged for 12 min at six frames per minute (fpm), whereas ANXA7-mRFP transfected cells were imaged for 0–3 min at one fpm, 4–6 min at two fpm, 7–10 min at six fpm and 11–15 min at one fpm to reduce photobleaching of the ANXA7-mRFP construct. MCF7 EGFP-LC3 cells transiently transfected with ANXA7-mRFP and siRNA transfected MCF7 cells transiently transfected with EGFP-LGALS3 and ANXA7-mRFP were imaged for 0–3 min at one fpm, 4–9 min at two fpm, 10–15 min at six fpm and 16–21 min at two fpm. LLOMe was added to a final concentration of 5 mM following the initial frame in all time-lapse videos.

### Magic red assay

MCF7-p95ErbB2 CRISPR-A7KO and CRISPR-Ctrl cells were transfected with AllStar Negative Ctrl or CHMP4B#2 siRNA according to the description in “Plasmids and siRNA transfections” and reseeded in a SCREENSTAR 96-well microplate (Greiner Bio-One, 655866) 24 h prior to the assay. Cells were incubated with 0.6 µM Sir-tubulin (Spirochrome, SC002) and 0.75 µM Nuclear Violet™ (AAT Bioquest, LCS1) for 1 h at 37°C before initiating the assay. Both dyes were present in the CIM throughout the entire assay. Magic Red (Immunochemistry Technology, 937) was reconstituted in DMSO according to the manufacturer’s instructions and stored at −80°C in single-use aliquots. For each experiment, aliquots were thawed immediately before use and diluted 1:2,500 in CIM, after which the cells were incubated with Magic Red solution for 3 min at 37°C before initiating the assay. Images were captured every minute at two sites in each well using an ImageXpress Micro Confocal High-Content Imaging System equipped with a CFI Apochromat LWD Lambda S 40XC/1.15 WI objective and 405/20 nm, 555 nm and 638/17 nm lasers and MetaXpress High Content Image Acquisition & Analysis Software (v6.6). Two frames were captured, and the acquisition paused. LLOMe was added to a final concentration of 1.5 mM, and imaging was resumed for 3 min. Following 3 min, the acquisition was paused, and cells were washed in clean CIM and administered CIM containing all three dyes before resuming imaging for another 9 min. Cells were kept at 37°C and supplied with 5% CO_2_ throughout all incubation periods and the entire assay.

Magic Red puncta were quantified by detecting puncta objects based on fluorescent intensity and pixel size within a defined cell. Top-hat filtering was included to reduce artifacts from fluorescent accumulation at object borders. The analysis output was the number of Magic Red puncta per cell at each timepoint normalized to the number of Magic Red puncta per cell in the first frame of the treatment condition.

### Cell death assay

Cells were treated with LLOMe at different concentrations (0–5 mM) and time intervals (0–120 min). To assess the dead/live ratio, cells were stained for 5 min at 37°C with a propidium iodide (Sigma-Aldrich, P4864, 0.25 μg/mL) and Hoechst 33342 (Sigma-Aldrich, 861405, 6.25 μg/mL) dye mix. The fluorescent intensities were measured using a Celigo^®^ Imaging Cytometer (Brooks Life Science Systems) and associated software (v4.1.3.0). The cytometer was equipped with a proprietary F-theta lens and 377/50 nm and 531/40 nm lasers.

### Lipid extraction and lipidomics

A modified Bligh and Dyer protocol was used for lipid extraction (Bligh and Dyer, 1959). Briefly, purified samples were resuspended in 200 µL 155 mM ammonium bicarbonate. 1 mL chloroform/methanol 2:1 (*v/v*) and 12.5 µL internal lipid standard mix was added (see Supplementary Table S2 for a list of all internal lipid standards used in this study). Samples were shaken at 2,000 rpm for 15 min at 4°C and centrifuged at 2,000 × g for 2 min at 4°C after which the upper phase was removed and 100 µL methanol and 50 µL 155 mM ammonium bicarbonate was added. Samples were shaken at 2,000 rpm for 1 min at 4°C and centrifuged at 2,000 × g for 2 min at 4°C. The lower phase was transferred to a new tube and solvents were evaporated for 75 min in a vacuum centrifuge. Samples were resuspended in 100 µL chloroform/methanol 1:2 (*v/v*), shaken at 2,000 rpm for 5 min at 4°C and spun down at 15,000 × g for 5 min at 4°C. Samples were loaded in positive (13.3 mM ammonium acetate in isopropanol) or negative [0.2% methyladenine (*v/v*) in 100% methanol] solvent. Samples were directly infused and analyzed in a positive and negative mode using the Orbitrap Fusion Tribrid (Thermo Fisher Scientific, San Jose, CA, United States) coupled to a TriVersa NanoMate (Advion Biosciences, NY, United States). The mass spectra were analyzed using a python-based software called LipidXplorer (Herzog et al., 2012). Using an in-house built R-based software, LipidQ (https://github.com/ELELAB/lipidQ), the absolute quantities were analyzed. The intensity values of the lipids were compared to their internal lipid standard to determine the absolute molar quantities of the lipids (see Supplementary Table S2 for a list of the internal lipid standards used in this study).

### Lipidomics data analysis

Lipid nomenclature was described previously (Nielsen I. O. et al., 2020). R statistical software [R Core Team (2021)] version 1.4.1717 was used to analyze the data. Lipids with a median quantity of all replicates below 0.0001 mol% were excluded from the analysis. To statistically analyze every lipid species or class, the “limma” package (Ritchie et al., 2015) was used. A linear model was fitted based on the quantity of the lipids. Log2-transformed fold change values and associated *p*-values were calculated. The Benjamini-Hochberg method was used to account for the false discovery rate (FDR) by correcting for multiple testing and adjusted *p*-values (*q*-values) were calculated. Lipids were considered statistically significantly changed if the *q*-values were below 0.05.

### Statistical analysis

Statistical analyses were conducted using an unpaired two-tailed *t*-test with Welch’s correction (α = 0.05) in the GraphPad Prism software (v9.0.0). All analyses were performed on the mean values from at least three independent experiments, except for the Magic Red assay. For the Magic Red assay, we quantified the area under the curve (AUC) from the last time point of LLOMe treatment until the endpoint, and the statistical analysis was based on the mean AUC from four independent experiments. Outliers were identified using the robust regression and outlier removal (ROUT) method (Q = 1%) for the number of CHMP4B and GAL3 puncta per cell in Figures 3A, B, D–G. Significance levels are denoted as follows: *p ≤ 0.05; **p ≤ 0.01; ***p ≤ 0.001; ****p ≤ 0.0001.

## Data Availability

The original contributions presented in the study are included in the article/Supplementary Material, further inquiries can be directed to the corresponding author.
