# Supplementary material for: Annexin A7 mediates lysosome repair independently of ESCRT-III
Source: Front Cell Dev Biol. 2024 Jan 23;11:1211498. doi: 10.3389/fcell.2023.1211498 (PMC10860759; doi:10.3389/fcell.2023.1211498)
Supplement: Supplementary file 6 [file DataSheet1.PDF]

Supplementary Material for

Annexin A7 mediates lysosome repair independently of ESCRT-III

Malene Laage Ebstrup†, Stine Lauritzen Sønder †, Ditte Louise Fogde, Anne Sofie Busk Heitmann, Tiina Naumanen Dietrich, Catarina Dias, Marja Jäättelä, Kenji Maeda & Jesper Nylandsted*

†Equal contribution

*Corresponding author email: jnl@cancer.dk

The PDF file includes:

Fig. S1-8
Table S1-2
Legends for movies S1-7

Other Supplementary Material for this manuscript includes the following:

Movies S1-7

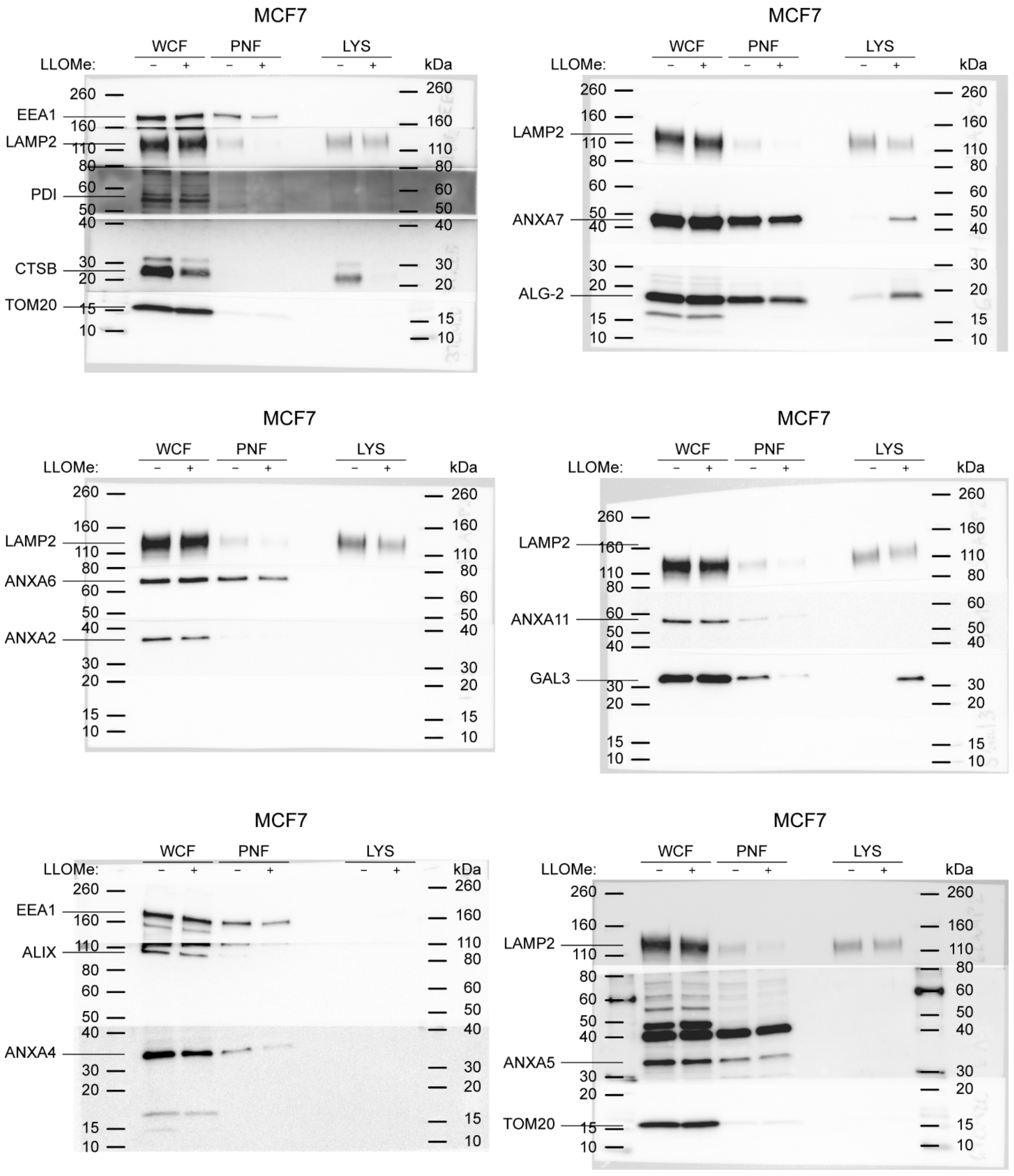
MCF7
WCF
PNF
LYS
LLOMe:
– + – + – +
kDa
EEA1
LAMP2
PDI
CTSB
TOM20
260
160
110
80
60
50
40
30
20
15
10
MCF7
WCF
PNF
LYS
LLOMe:
– + – + – +
kDa
LAMP2
ANXA7
ALG-2
MCF7
WCF
PNF
LYS
LLOMe:
– + – + – +
kDa
LAMP2
ANXA6
ANXA2
MCF7
WCF
PNF
LYS
LLOMe:
– + – + – +
kDa
LAMP2
ANXA11
GAL3
MCF7
WCF
PNF
LYS
LLOMe:
– + – + – +
kDa
EEA1
ALIX
ANXA4
MCF7
WCF
PNF
LYS
LLOMe:
– + – + – +
kDa
LAMP2
ANXA5
TOM20

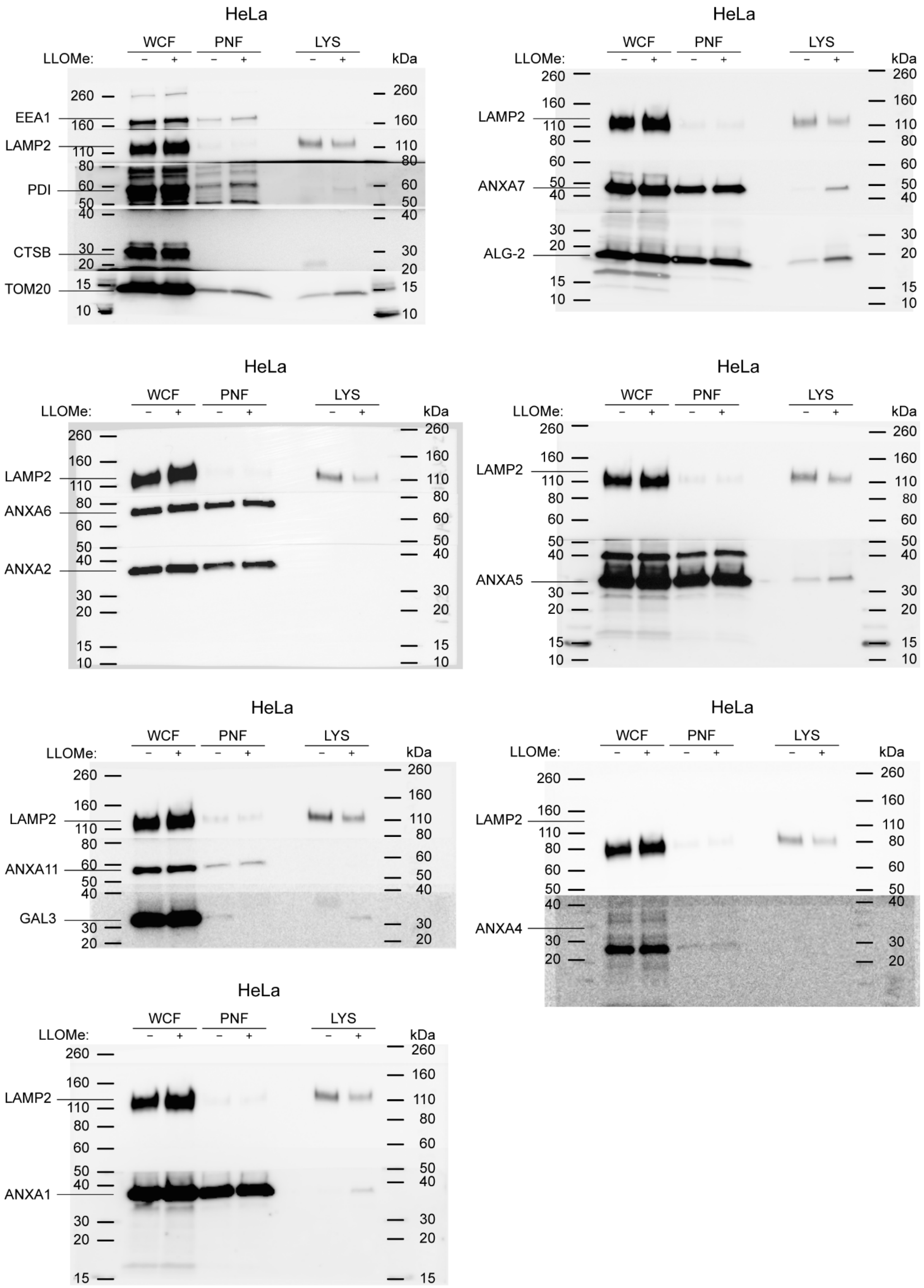
HeLa
WCF
PNF
LYS
LLOMe:
kDa
EEA1
LAMP2
PDI
CTSB
TOM20
ANXA7
ALG-2
ANXA6
ANXA2
ANXA5
ANXA11
GAL3
ANXA4
ANXA1
260
160
110
80
60
50
40
30
20
15
10

Figure S1. (Related to Fig. 1C, E, G, H). **Unedited immunoblots from FeDEX-mediated lysosomal isolation.** Immunoblot of WCF, PNF and LYS fractions from MCF7 and HeLa cells treated with vehicle (diH_2O) or LLOMe (2 mM) for 1 h showing bands detected with antibodies against the indicated organelle compartments. Abbreviations: (ALG2) apoptosis-linked gene-2, (ANXA) annexin, (CTSB) cathepsin B, (EE) early endosome, (EEA1) early endosome antigen 1, (FeDEX) superparamagnetic colloidal iron dextran, (GAL3) galectin-3, (LAMP2) lysosome-associated membrane glycoprotein 2, (LLOMe) L-Leucyl-L-Leucine *O*-methyl ester, (LYS) lysosome, (PDI) protein disulfide-isomerase, (PNF) post-nuclear fraction, (TOM20) mitochondrial import receptor subunit TOM20, (WCF) whole-cell fraction.

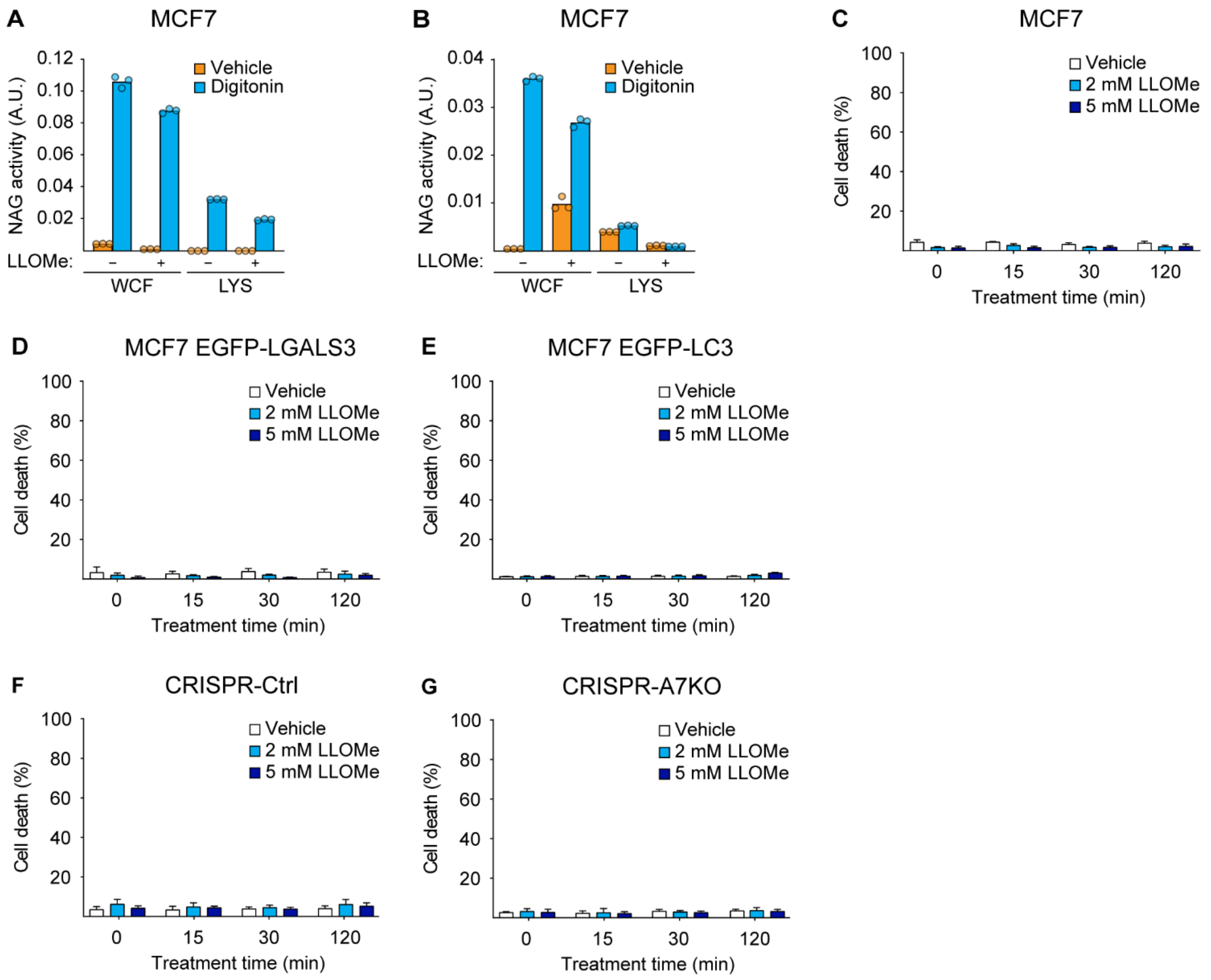

Figure S2. (Related to Fig. 1D, 2). **NAG activity and cell death assays.** (A-B) NAG activity of WCF and LYS fractions after FeDEX-based isolation from the remaining biological replicates. The NAG activity was used to indirectly assess LLOMe-induced LMP using the membranolytic agent, digitonin, as a positive control. Data points reflect technical replicates. (C-G) Cell death (%) of MCF7, MCF7 EGFP-LGALS3, MCF7 EGFP-LC3, MCF7-p95ErbB2 CRISPR-Ctrl and CRISPR-A7KO cells after 0, 15, 30 and 120 min exposure to vehicle (diH_2O) or LLOMe at the indicated concentrations. Data are mean ± SD from three independent experiments. Abbreviations: (NAG) N-acetyl-β-D-glucosaminidase. Remaining abbreviations as in figure S1.

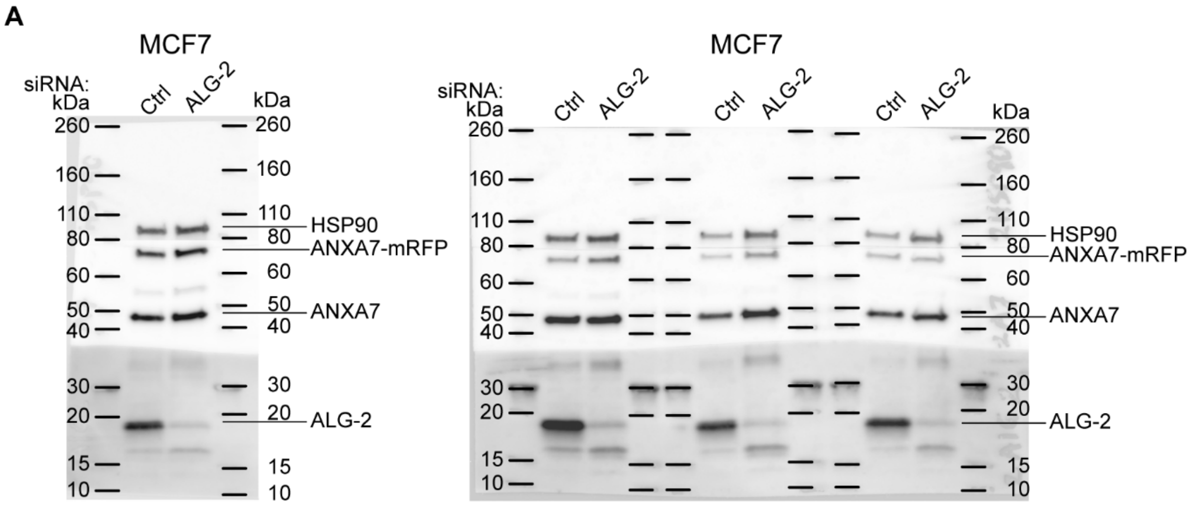

Figure S3. (Related to Fig. 2D). **Unedited immunoblots from ANXA7 puncta formation after ALG-2 siRNA transfection.** (A) Immunoblot of lysates from MCF7 transiently co-expressing EGFP-Gal3 and ANXA7-mRFP and transfected with Ctrl or ALG-2 siRNA (72h) showing bands detected with antibodies against ALG-2, ANXA7 and HSP90 (loading control). Abbreviations as in figure S1.

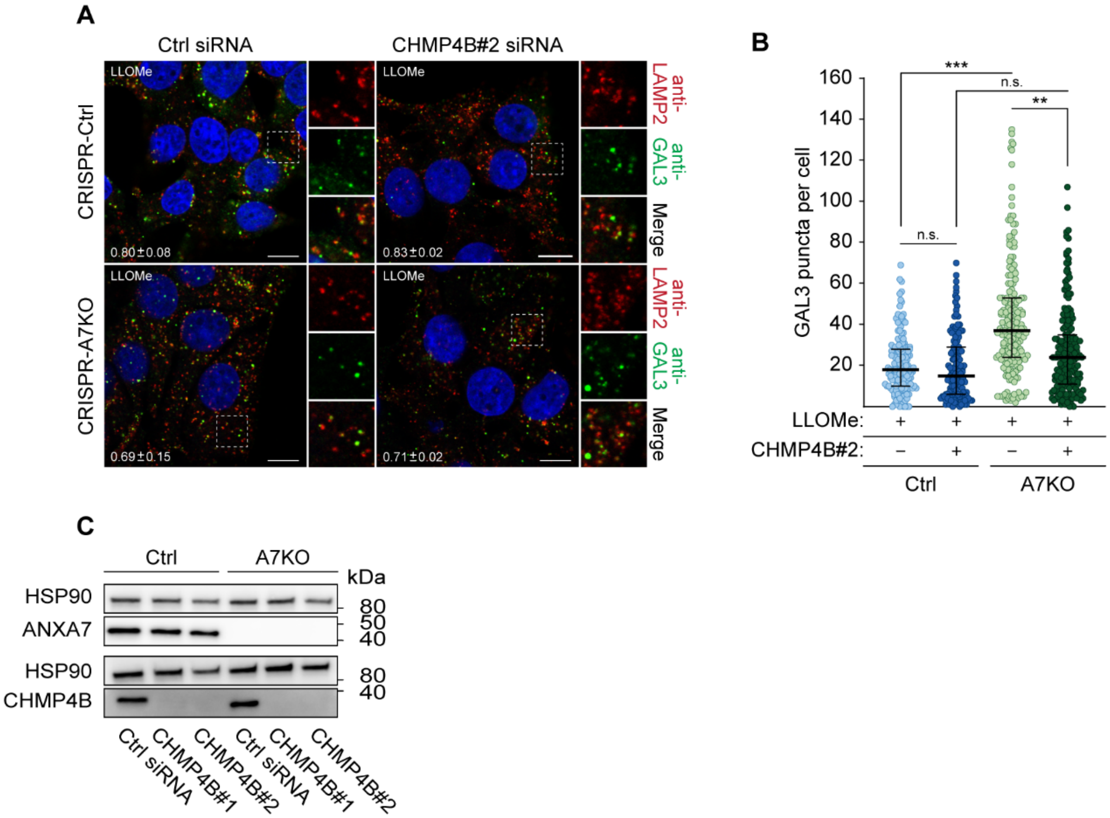

Figure S4. (Related to Fig. 3). **ANXA7 knockout sensitizes MCF7-p95ErbB2 cells to LLOMe-induced lysosomal membrane permeabilization.** (A) Representative images MCF7-p95ErbB2 CRISPR-A7KO and CRISPR-Ctrl cells transfected with Ctrl or CHMP4B#2 siRNA (72 h) and treated LLOMe (2 mM, 30 min) prior to fixation and immunostaining with Hoechst 33342 (blue), anti-GAL3 (green) and anti-LAMP2 (red). (B) GAL3 puncta per cell quantified from the data described in (A). Data from four independent experiments are quantified from ≥ 28 cells per condition and depicted as single observations, median and interquartile range. (C) Immunoblot of lysates from MCF7-p95ErbB2 CRISPR-A7KO and CRISPR-Ctrl cells transfected with Ctrl, CHMP4B#1 or CHMP4B#2 siRNA showing bands detected with antibodies against ANXA7, CHMP4B and HSP90 (loading control). Original unedited blots are presented in supplementary Fig. S5C-D. The mean Manders' overlap coefficient ± SD is presented in the bottom left corner of each image (A). The p-values were defined by an unpaired two-tailed t-test with Welch's correction comparing the mean values between the indicated conditions. Scale bar denotes 10 µm. **P ≤ 0.01. Abbreviations: (CHMP4B) charged multivesicular body protein 4B, (HSP90) heat shock protein 90. Remaining abbreviations as in figure S1.

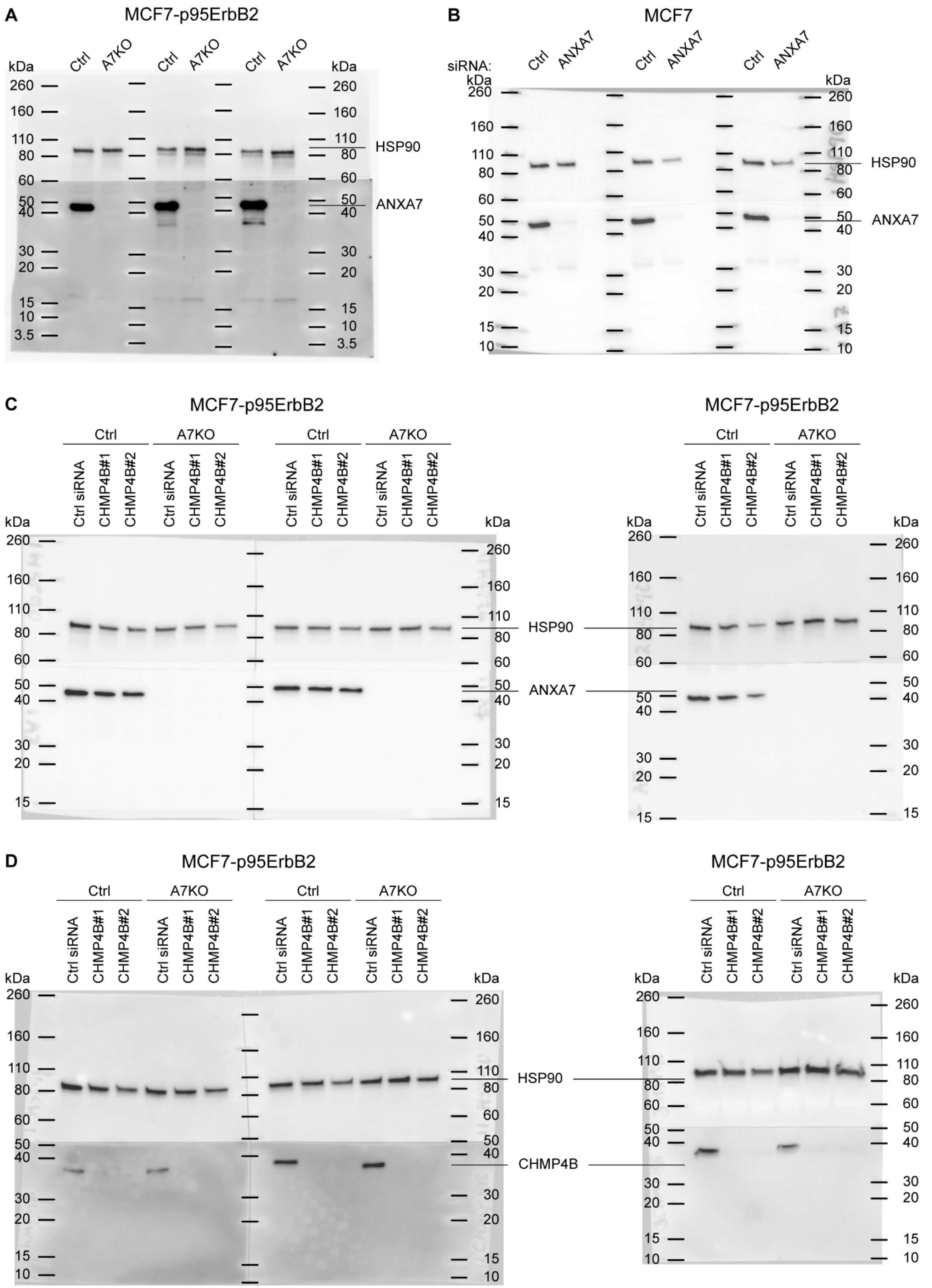
A
MCF7-p95ErbB2
Ctrl
A7KO
kDa
HSP90
ANXA7
B
MCF7
siRNA:
Ctrl
ANXA7
kDa
HSP90
ANXA7
C
MCF7-p95ErbB2
Ctrl
A7KO
Ctrl siRNA
CHMP4B#1
CHMP4B#2
kDa
HSP90
ANXA7
MCF7-p95ErbB2
D
MCF7-p95ErbB2
Ctrl
A7KO
Ctrl siRNA
CHMP4B#1
CHMP4B#2
kDa
HSP90
CHMP4B
MCF7-p95ErbB2

Figure S5. (Related to Fig. 3C, H, S4). **Unedited immunoblots from CHMP4B and GAL3 staining.** (A) Immunoblot of MCF7-p95ErbB2 CRISPR-A7KO and CRISPR-Ctrl lysates showing bands detected with antibodies against ANXA7 and HSP90 (loading control). (B) Immunoblot of lysates from MCF7 cells transfected with Ctrl or ANXA7 siRNA showing bands detected with antibodies against ANXA7 and HSP90 (loading control). (C-D) Immunoblot of lysates from MCF7-p95ErbB2 CRISPR-A7KO and CRISPR-Ctrl cells transfected with Ctrl, CHMP4B#1 or CHMP4B#2 siRNA (72 h) showing bands detected with antibodies against ANXA7 (C), CHMP4B (D) and HSP90 (loading control) (C-D) (Fig. S4). Abbreviations as in figures S1 and S3.

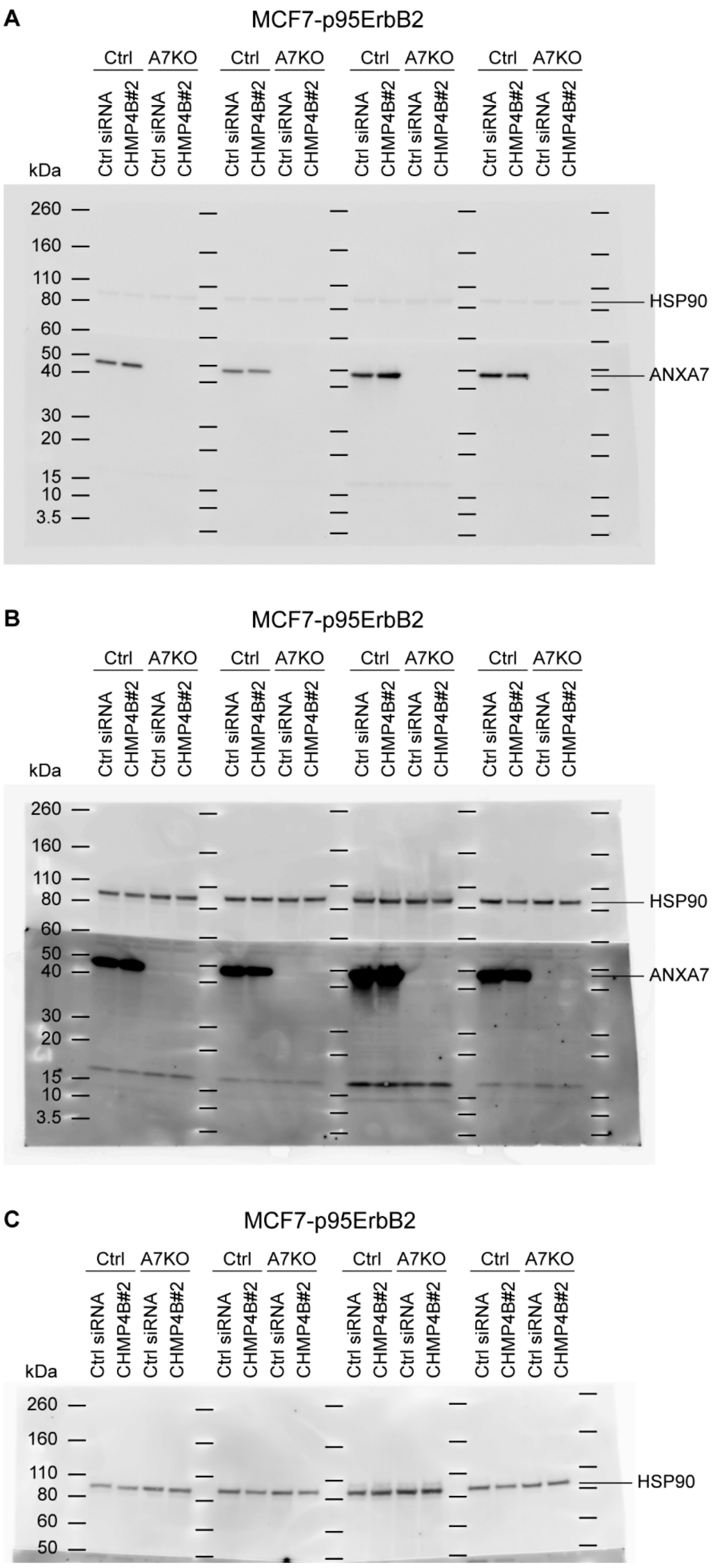
A
MCF7-p95ErbB2
Ctrl
A7KO
Ctrl siRNA
CHMP4B#2
kDa
260
160
110
80
60
50
40
30
20
15
10
3.5
HSP90
ANXA7
B
MCF7-p95ErbB2
Ctrl
A7KO
Ctrl siRNA
CHMP4B#2
kDa
260
160
110
80
60
50
40
30
20
15
10
3.5
HSP90
ANXA7
C
MCF7-p95ErbB2
Ctrl
A7KO
Ctrl siRNA
CHMP4B#2
kDa
260
160
110
80
60
50
HSP90

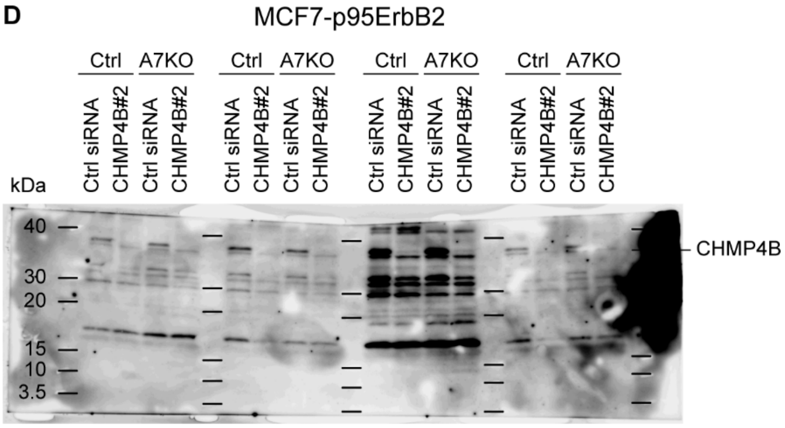

Figure S6. (Related to Fig. 4C). **Unedited immunoblots from the Magic Red assay.** (A-D) Immunoblot of lysates from MCF7-p95ErbB2 CRISPR-A7KO and CRISPR-Ctrl cells transfected with Ctrl or CHMP4B#2 siRNA (72 h) showing bands detected with antibodies against ANXA7 (A-B), CHMP4B (D) and HSP90 (loading control) (A-C). Abbreviations: same as in figures S1 and S3.

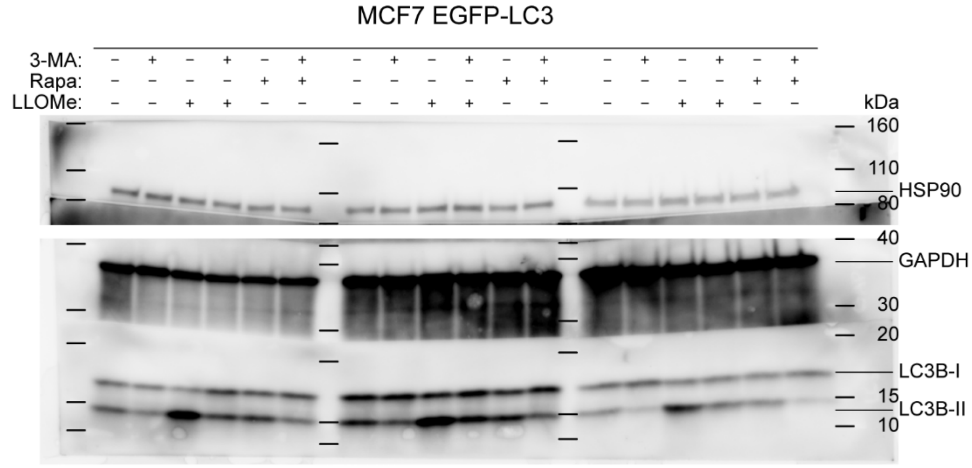

Figure S7. (Related to Fig. 5B). **Unedited immunoblots confirming induction or inhibition of autophagy.** Immunoblot of lysates from MCF7 EGFP-LC3 cells treated with vehicle (cell culture medium), 3-MA (10 mM, 30 min), LLOMe (5 mM, 15 min) or rapamycin (200 nM, 2 h) showing bands detected with antibodies against HSP90 (loading control), GAPDH (loading control) and LC3. Abbreviations: (3-MA) 3-methyladenine, (GAPDH) glyceraldehyde-3-phosphate dehydrogenase, (LC3) microtubule-associated protein 1A/1B-light chain 3. Remaining abbreviations as in figures S1 and S3.

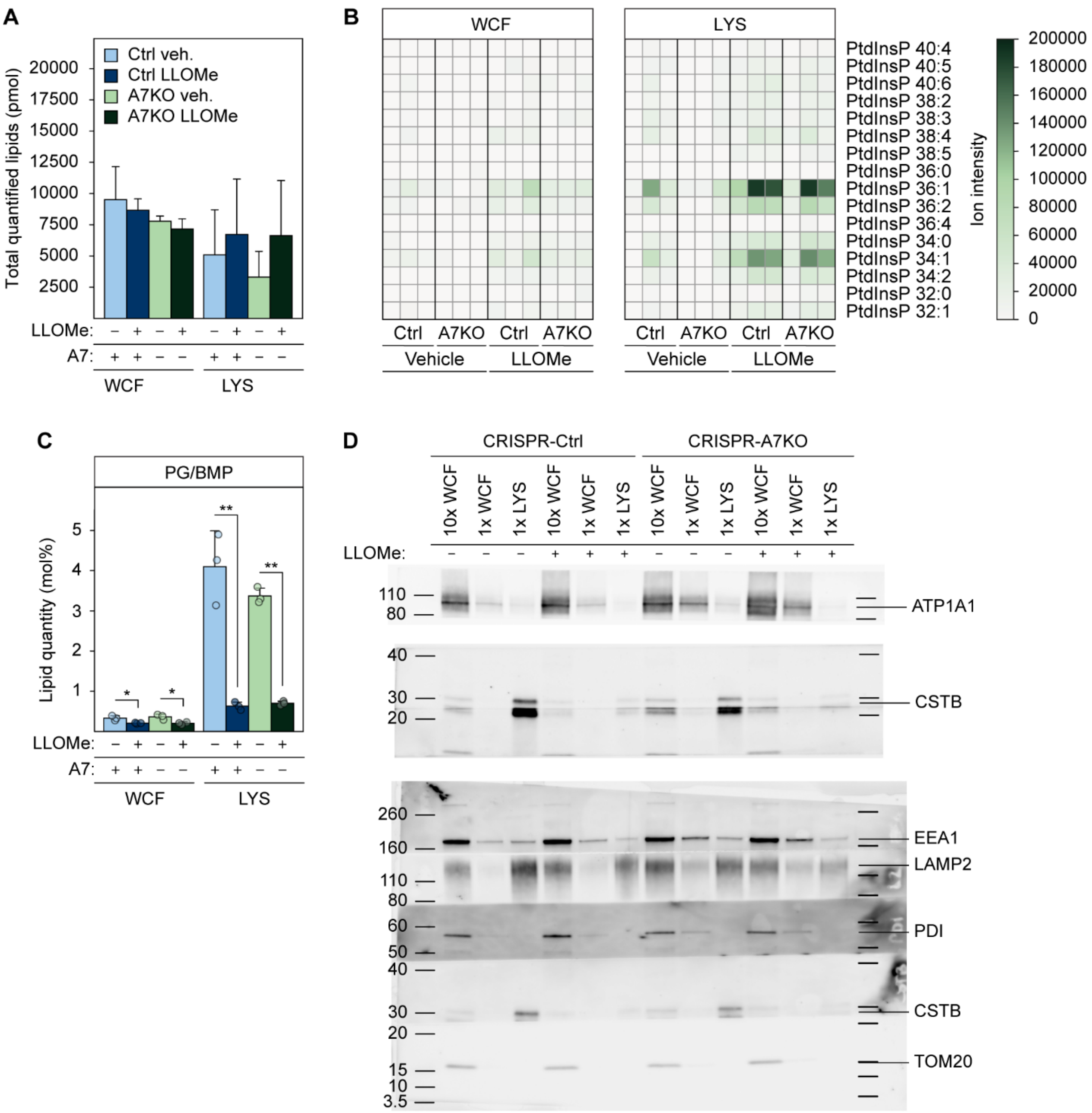

Figure S8. (Related to Fig. 6). **Supplementary lipidomics data and unedited immunoblots.** WCF or purified LYS from MCF7-p95ErbB2 CRISPR-A7KO and CRISPR-Ctrl cells treated either with vehicle (diH_2O) or LLOMe (2 mM) for 1 h. (A) Total molar quantity of lipids identified according to treatment. (B) Ion intensities of PtdInsPs detected in the MS2 spectra. Values from each replicate are depicted in separate rows. (C) Levels of PG/BMP given in mol%. Circles represent individual replicates. Linear modeling with a Benjamini-Hochberg correction was used to determine statistical significance in (C). *P ≤ 0.05; **P ≤ 0.01. (D) Immunoblot of WCF and LYS lysates with protein markers of the indicated organelle compartments. Loading was adjusted according to the total lipid content. Abbreviations: (ATP1A1) sodium/potassium-transporting ATPase subunit alpha-1, (BMP) bis(monoacylglycero)phosphate, (PG) phosphatidylglycerol, (PtdInsP) phosphatidylinositol phosphate. Remaining abbreviations as in figure S1.

Table S1. (Related to the Materials and Methods section). **Antibody information for Western blotting.**

Antibody	Supplier	Identifier	Dilution
ALG-2 (PDCD6)	Abcam #ab133326	RRID:AB_300242	1:1000
ALIX	Cell signaling #2171	RRID:AB_2299455	1:1000
ANXA1	Abcam #65844	RRID:AB_1139408	1:500
ANXA2	BD Biosciences #610069	RRID:AB_397480	1:1000
ANXA4	R&D Systems #MAB4146	RRID:AB_10892100	1:200
ANXA5	Abcam #ab14196	RRID:AB_300979	1:1000
ANXA6	Abcam, #ab31026	RRID:AB_722810	1:1000
ANXA7	Abcam #ab49838<br>Termo Fisher Scientific #PA5-35358	RRID:AB_2274155<br>RRID:AB_2552668	1:1000<br>1:1000
ANXA11	Abcam #ab166846	-	1:1000
ATP1A1	Abcam #76020	RRID:AB_1310695	1:10,000
CHMP4B	Abcam #ab105767	RRID:AB_10858466	1:1000
CTSB	Sigma-Aldrich #C6243<br>Cell signaling #31718	RRID:AB_1078403<br>RRID:AB_2687580	1:1000<br>1:1000
EEA1	Abcam #ab2900	RRID:AB_2148422	1:1000
GAL3	BD Biosciences #556904	RRID:AB_396531	1:1000
**GAPDH**	Abcam # ab189095	-	1:7500
HSP90	BD Biosciences #610418	RRID:AB_397798	1:4000
LAMP2	DSHB #H4B4	RRID_AB:528129	1:1000
**LC3B**	Cell signaling #3868	RRID_AB:2137707	1:1000
PDI	Abcam #ab3672<br>Abcam #ab137110	RRID:AB_303990<br>RRID:AB_2915914	1:1000<br>1:1000
TOM20	Santa Cruz Biotechn #SC-11415	RRID:AB_2207533	1:3000
Rabbit IgG (goat) HRP-conjugated	Vector Laboratories #PI-1000	RRID:AB_2336198	1:5000
Mouse IgG (rabbit) HRP-conjugated	Dako #P0450	RRID:AB_2630354	1:5000

Table S2. (Related to Fig. 6). **Internal lipid standards.** (CE) cholesteryl ester, (Cer) ceramide, (CerP) Cer phosphate, (Chol) cholesterol, (CL) cardiolipin, (DAG) diacylglycerol, (FA) fatty acid, (GM3) ganglioside GM3, (HexCer) hexosylceramide, (diHexCer), dihexosylceramide, (triHexCer) trihexosylceramide, (LHexCer) lysohexosylceramide, (PA) phosphatidic acid, (LPA) lysoPA, (PC) phosphatidylcholine, (LPC) lysoPC, (PE) phosphatidylethanolamine, (LPE) lysoPE, (LPG) lysoPG, (PI) phosphatidylinositol, (LPI) lysoPI, (PS) phosphatidylserine, (LPS) lysoPS, (FA) fatty acid, (LCB) long-chain base, (LCBP) LCB phosphate, (LSM) lysosphingomyelin, (SHexCer) sulfatide, (TAG) triacylglycerol. Remaining abbreviations as in figure S6.

Lipid class	Standard	Molar quantities (pmol)
CE	CE 15:0-D7	45.80
Cer	Cer 18:1;2/12:0;0	31.25
Chol	Chol-D4	356.88
DAG	DAG 12:0-12:0	45.63
HexCer	HexCer18:1;2/12:0;0	28.86
LPA	LPA 17:0	31.25
LPG	LPG 13:0	11.13
LPI	LPI 13:0	22.88
LPS	LPS 17:1	14.25
PA	PA 12:0-12:0	28.38
PE	PE 12:0-12:0	39.45
PG	PG 12:0-12:0	29.38
PI	PI 8:0-8:0	28.39
PS	PS 12:0-12:0	25.35
SM	SM 18:1;2/12:0;0	21.25
TAG	TAG 17:0/17:0/17:0	907.50
PC	PC 12:0-12:0	36.00
LPE	LPE 17:1	30.79
LPC	LPC 12:0	25.00
diHexCer	diHexCer18:1;2/17:0;0	23.25
BMP	BMP 28:0	25.00
CerP	CerP 18:1;2/12:0;0	25.00
LCB	LCB 17:1;2	25.00
LCBP	LCBP 17:1;2	25.00
LSM	LSM 17:1;2	25.00
SHexCer	SHexCer18:1;2/12:0;0	35.78
triHexCer	triHexCer 18:1;2/17:0;0	51.25
LHexCer	LHexCer 13C6 18:1;2	25.00
CL	CL 14:0	72.19
FA	FA 16:0-D4	37.50
GM3	GM3 36:1;2-D3	37.50

Movie S1. (Related to Fig. 2A). **ANXA7 translocates to GAL3-positive vesicles following LLOMe-induced lysosomal membrane permeabilization independently of ALG-2.** GAL3 and ANXA7 puncta formation in response to LLOMe (5 mM) treatment in MCF7 cells transfected with control siRNA and transiently co-expressing EGFP-GAL3 and ANXA7-mRFP. Abbreviations as in figure S1.

Movie S2. (Related to Fig. 2B). **ANXA7 translocates to GAL3-positive vesicles following LLOMe-induced lysosomal membrane permeabilization independently of ALG-2.** GAL3 and ANXA7 puncta formation in response to LLOMe (5 mM) treatment in MCF7 cells transfected with ALG-2 siRNA and transiently co-expressing EGFP-GAL3 and ANXA7-mRFP. Abbreviations as in figure S1.

Movie S3. (Related to Fig. 2E). **ANXA7 translocates to GAL3-positive vesicles following LLOMe-induced lysosomal membrane permeabilization.** GAL3 and ANXA7 puncta formation in response to LLOMe (5 mM) in MCF7-EGFP-LGALS3 cells transiently co-expressing ANXA7-mRFP. Abbreviations as in figure S1.

Movie S4. (Related to Fig. 2F). **ANXA2 remains unresponsive following LLOMe-induced lysosomal membrane permeabilization.** GAL3 puncta formation is observed in MCF7-EGFP-LGALS3 cells transiently co-expressing ANXA2-mRFP in response to LLOMe (5 mM), while ANXA2 maintains a uniform distribution across the cytoplasm. Abbreviations as in figure S1.

Movie S5. (Related to Fig. 2G). **ANXA5 remains unresponsive following LLOMe-induced lysosomal membrane permeabilization.** GAL3 puncta formation is observed in MCF7-EGFP-LGALS3 cells transiently co-expressing ANXA5-tRFP in response to LLOMe (5 mM), while ANXA5 maintains a uniform distribution across the cytoplasm. Abbreviations as in figure S1.

Movie S6. (Related to Fig. 5D). **LC3 and ANXA7 puncta do not coincide upon LLOMe-induced lysosomal membrane permeabilization.** In MCF7 EGFP-LC3 cells transiently co-expressing ANXA7-mRFP and pretreated with vehicle (cell culture medium, 30 min), LC3 and ANXA7 puncta were observed at different cellular locations in response to LLOMe (5 mM). Abbreviations: (3-MA) 3-methyladenine, (LC3) microtubule-associated protein 1A/1B-light chain 3 Remaining abbreviations as in figure S1

Movie S7. (Related to Fig. 5E). **Blocking lysophagy fails to prevent ANXA7 puncta formation in response to LLOMe-induced lysosomal membrane permeabilization.** Pretreating MCF7 EGFP-

LC3 transiently co-expressing ANXA7-mRFP with the generic autophagy inhibitor 3-MA (10 mM, 30 min) markedly reduced LC3 puncta formation upon LLOMe (5 mM) treatment. However, ANXA7 puncta formation persists to a similar extent as observed with vehicle treatment (Fig. 5B, E and Movie S6), underscoring the independent function of ANXA7 from lysophagy. The absence of co-occurring LC3 and ANXA7 further supports this conclusion. Abbreviations: (3-MA) 3-methyladenine, (LC3) microtubule-associated protein 1A/1B-light chain 3 Remaining abbreviations as in figure S1
